# Single-cell RNA sequencing reveals a distinct profile of bone immune microenvironment and decreased osteoclast differentiation in type 2 diabetic mice

**DOI:** 10.1016/j.gendis.2023.101145

**Published:** 2023-10-17

**Authors:** Zimei Wu, Qiaodan Hou, Heng Chi, Jihong Liu, Yixin Mei, Tingting Chen, Kunkun Yang, Jingna Zheng, Jing Xu, Fuxin Wei, Lin Wang

**Affiliations:** a School of Medicine, Southern University of Science and Technology, Shenzhen, Guangdong 518055, China; b Southern University of Science and Technology Hospital, Shenzhen, Guangdong 518055, China; c Department of Orthopedic Surgery, The Seventh Affiliated Hospital of Sun Yat-sen University, Shenzhen, Guangdong 518107, China

**Keywords:** AP-1, Bone immune microenvironment, Osteoclast differentiation, Single-cell RNA sequencing, Type 2 diabetes

## Abstract

The pathogenic effects of type 2 diabetes on bone tissue are gaining attention, but the cellular and molecular mechanisms underlying osteoimmunology are still unclear in diabetes-related bone diseases. We delineated the single-cell transcriptome of bone marrow cells from both wide type and type 2 diabetes mice, which provided the first detailed global profile of bone marrow cells and revealed a distinct bone immune microenvironment at the genetic level under type 2 diabetic condition. It was observed that osteoclast activity was inhibited due to a dysregulated cytokine network, which ultimately led to decreased osteoclast formation and differentiation. In type 2 diabetes mice, a specific *Cd36*^*+*^ cluster (cluster 18, monocytes/macrophages 2) was identified as the precursor of osteoclasts with diminished differentiation potential. *AP-1* was demonstrated to be the key transcription factor in the underlying mechanism.

## Introduction

Diabetes influences at least 537 million people globally, of which type 2 diabetes (T2D) accounts for over 90%.^1^ As a systemic metabolic disease, T2D can affect multiple tissues and organs, such as the heart, blood vessels, eyes, kidneys and nerves, and induce more than 100 complications, which are the main causes of morbidity and mortality in the diabetic population.^2^^,^^3^ However, the impact of diabetes on the skeleton system is often overlooked and underestimated, and the clinical appearance of diabetic bone disease, a particular chronic complication, is often insidious unless in the T2D patient with a long course duration. As the high prevalence of T2D and the rapidly increasing incidence of youth-onset T2D (aged 45 years or younger),^1^^,^^4^ more and more patients are exposed to a long course duration and the threat of diabetic bone disease, which involves a high risk of fracture (RR = 1.38; 95% CI: 1.25–1.53),^5^ delayed bone healing, and a high recurrent fracture rate.^6^^,^^7^

Bone marrow (BM) is the major site of hematopoiesis, which provides a unique microenvironment of niches mainly supporting self-renewal and differentiation of hematopoietic stem cells, myeloid and lymphoid progenitors, and mature immune cells.^8^ BM is also one of the human central immune organs; bone cells (including osteoclasts, osteoblasts, osteocytes, *etc*.) and immune cells co-exist in the BM microenvironment. They not only have common progenitors but also share a diversity of regulatory molecules, including cytokines, receptors, and hormones, which further communicate with each other to cooperatively perform the functions of the “osteoimmune system”.^9^ Under a physiological environment, the intercommunication between bone cells and immune cells maintains the bone balance.^10^ Several biostudies in mice, rats, and humans demonstrate that diabetes results in multiple BM microenvironmental defects (microangiopathy^11^^,^^12^ and neuropathy^13^) and damaged stem cell mobilization (mobilopathy^14^, ^15^, ^16^). Numerous researches have reported the effects and underlying mechanisms of T2D conditions on bone remodelling including bone formation and bone resorption. However, the influence of diabetes on osteoimmunology and bone metabolism in the BM microenvironment remains unclear.

Single-cell RNA sequencing (scRNA-seq), as an objective and effective method, plays an irreplaceable role in characterizing diverse cell types in complicated tissues in physiological and pathological environments.^17^ To comprehensively understand the impact of the T2D pathological condition on osteoimmunology and bone metabolism in the BM microenvironment, we carried out high-resolution scRNA-seq to capture T2D-induced gene alterations in 14,599 individual BM cells from a typical T2D mouse model with the hallmark characteristics of adult-onset T2D in humans.^18^ To the best of our knowledge, this is the first systematic depiction of BM cells under T2D conditions using scRNA-seq, providing unique insights regarding changes in medullary cells and microenvironmental cytokines, a specific cell cluster and trend of osteoclast inhibition in T2D mice.

## Materials and methods

### T2D mice model

Male C57/BL6 mice (12 weeks old) were purchased from Jiangsu JicuiYaokang Biopharmaceutical Co., Ltd. Mice were housed in ventilated cages (23 °C) and maintained in a pathogen-free, accredited facility on a 12-h/12-h light/dark cycle. All assessments were performed in a blinded fashion. Mice were randomly assigned to the WT group and the T2D group. Mice in the T2D group were treated with a high-fat diet (Cat# D12492, Research Diets) and a single dose of intraperitoneal injection of streptozotocin (100 mg/kg, Cat# S0130, Sigma) to establish a T2D model.^18^ The specific protocol was as follows: 3-month-old C57BL/6 male mice were fed with a 1-month high-fat diet (lead–in phase) for high-fat diet-induced insulin resistance, and then given a single-dose intraperitoneal injection of streptozotocin (100 mg/kg in 50 mM sodium citrate buffer, pH 4.5, Sigma), followed by a 3-month high-fat diet (experimental phase). During the 3-month experimental period, non-fasting blood glucose values were obtained from the tail using a blood glucose monitor at the same time of day each week. High-fat diet/streptozotocin (*i.e.*, T2D) mice that did not reach a median glucose level above 13.8 mmol/L within 6 weeks after streptozotocin injection were excluded from the study.

BM from one mouse in each group aged 7 months was harvested from the femur and tibia for single-cell sequencing. The left femurs and tibias of mice in the WT group (*n* = 5, 7 months) and the T2D group (*n* = 5, 7 months) were used for micro-CT. In addition, the four right legs from each group were dissected for flow cytometry analysis. All experiments referring to animals were conducted in accordance with the guidelines of the Institutional Animal Care and Use Committee and approved by the Ethics Committee of Southern University of Science and Technology University (Approval No. SUSTech-JY202103018).

### Micro-CT analysis

All imaging and analysis were conducted in a blinded fashion. Computed tomographic images of the left femurs were acquired using a micro-CT scanner (Skyscan1276, Bruker) at high resolution. Scan settings were as follows: 60 kV source voltage, 100 μA source current, 20.3 μm pixel size, and 605 ms exposure time.^19^ After the femur was reconstructed, bone mineral density (BMD; mg/cm^3^), trabecular bone volume fraction (BV/TV; %), trabecular number (Tb.N), trabecular thickness (Tb.Th), and trabecular separation (Tb.Sp), were assessed using the manufacturer's software Sky Scan NRecon package, CT Analyser (Version 1.13).

### Bone marrow dissociation

Mice were anesthetized with isoflurane. The femur and tibia were dissected and the muscle and periosteum of the outer surface of the femur and tibia were removed. The bones were chopped into approximately 1 mm^3^ pieces and digested with 1 mg/mL collagenase I/II (Roche, Cat# 46793022). After 40 min incubation at 37 °C, the cell suspension was obtained by filtering the mixture through a 40 μm pore size filter, and centrifuged at 1500 g for 5 min. After resuspension in 1 × red blood cell lysis buffer (BioLegend, Cat# 420301) for 5 min to remove red blood cells, the cell suspension was centrifuged at 500 *g* for 5 min and resuspended in phosphate buffered saline solution. All the above steps were performed on ice except that the incubation at 37 °C was required.

### Single-cell RNA sequencing

Single-cell capture and cDNA synthesis were performed using the Single Cell 3′ Library and Gel Bead Kit V3 (10 × Genomics, 1000075) and Chromium Single Cell B Chip Kit (10 × Genomics, 1000074). Single-cell RNA-seq libraries were constructed using the Single Cell 3′ Library and Gel Bead Kit V3, according to the manufacturer's instruction, and quality verified. The libraries were sequenced by an Illumina Novaseq6000 sequencer and evaluated by FastQC and MultiQC.

### ScRNA-seq data pre-processing

The raw fastq files were used to align to the mouse genome reference sequence (mm10) and classify barcodes by Cell Ranger v2.2.0. The gene-cell expression matrix as an input file was used in Seurat (v 3.0). Low-quality cells (gene numbers <200 and mitochondrial genes >25%) were filtered, and the rest cells were employed in the following analysis.

### Bioinformatics analysis

Harmony removed batch effect between different samples. Classifying cell type was performed with the FindClusters function of Seurat in 47 PC and 0.6 resolution and annotated these cells based on marker genes with the CellMarker database and other published data.^20^ FindAllMarkers and FindMarkers functions of Seurat were used to perform differentially expressed analysis, and ClusterProfiler^21^ and KOBAS^22^ were used in the following function enrichment of differentially expressed genes (DEGs). Olcano plots, violin plots, and dot plots were generated with ggplot2. Trajectory analysis of cells that may have an evaluation relationship was performed by monocle 2.^23^ We also used FindMarkers to find DEGs between different states in pseudotime analysis result and function enrichment.

### Flow cytometry

To verify and sort *Cd36*^*+*^ cells in BM, we collected single-cell suspension of BM as described in scRNA-seq. After lysing and removing the red blood cells, the cells were washed and resuspended in staining buffer (BioLegend, Cat #420201), and incubated with Fc block (BioLegend, Cat#101302) for 10 min on ice to reduce non-specific immunofluorescent staining. Then, the cells were incubated with APC anti-mouse CD36 antibody (BioLegend, Cat# 102612, 1:50) for 45 min on ice. After washing, the cells were resuspended in phosphate buffered saline solution with 1 μg/mL DAPI (live/dead exclusion), and acquired on a FACS Canto flow cytometer (BD Biosciences). The data were analyzed using FlowJo software (Tree Star).

### Quantitative real-time PCR (qPCR)

*Cd36*^*+*^ BM cells were sorted directly into the lysis buffer of the RNA-easyTM Isolation Reagent (Vazyme, Cat# RC112-01) by a FACS (BD FACSAria SORP). The qPCR analysis was performed using standard procedure according to the manufacturer's instructions. For quantification, the mRNA of the cells was reversely transcribed into cDNAs with the PrimeScript™ RT Master Mix (Vazyme, Cat# R323-01). The resulting cDNAs were quantified with ChamQ SYBR Color qPCR Master Mix (Vazyme, Cat# Q712) to determine the mRNA levels of scRNA-seq identified and reported osteoclast differentiation-associated genes in sorted *Cd36*^*+*^ BM cells. All gene expression levels were normalized to endogenous *Actb*. The primer sequences are listed in Table S2.

### Osteoclast differentiation

The bone mononuclear macrophages were cultured with M-CSF (2 ng/μL) and RANKL (40 ng/μL) and then incubated at 37 °C, 7% CO_2_. After 4 days, TRAP was stained as described previously.^24^

### Actin ring assay

The bone mononuclear macrophages were cultured as described above. After four days, osteoclast actin rings were stained with AbFluor™ 488 phalloidin (Abbkine, Cat# BMD0082).

### Statistical analysis

Most of the statistical analyses were performed in R software. Data analyses were performed with GraphPad Prism 8. Data were presented as mean ± standard deviation. Significant differences in the mean values between the two groups were determined using Student's *t*-test. *P* < 0.05 was considered statistically significant.

## Results

### Identification of BM cells in T2D mice by scRNA-seq

We employed a classic T2D mice model induced with a high-fat diet and an intervention of streptozotocin starting in the adulthood of the mice. The HbA1C level (Fig. S1A), blood glucose level (Fig. S1B), and non-fasting body weight (Fig. S1C) were measured and the successful establishment of the T2D model was verified. The micro-CT images showed that the cortical bone was obviously thinner in T2D group and the trabecular bone of T2D group was significantly reduced (Fig. S1D), indicated by decreased BMD (*P* < 0.001), BV/TV (*P* < 0.01), Tb.N (*P* < 0.01), and Tb.Th (*P* < 0.05), as well as increased Tb.Sp (*P* < 0.05) (Fig. S1E).

**Figure 1:**
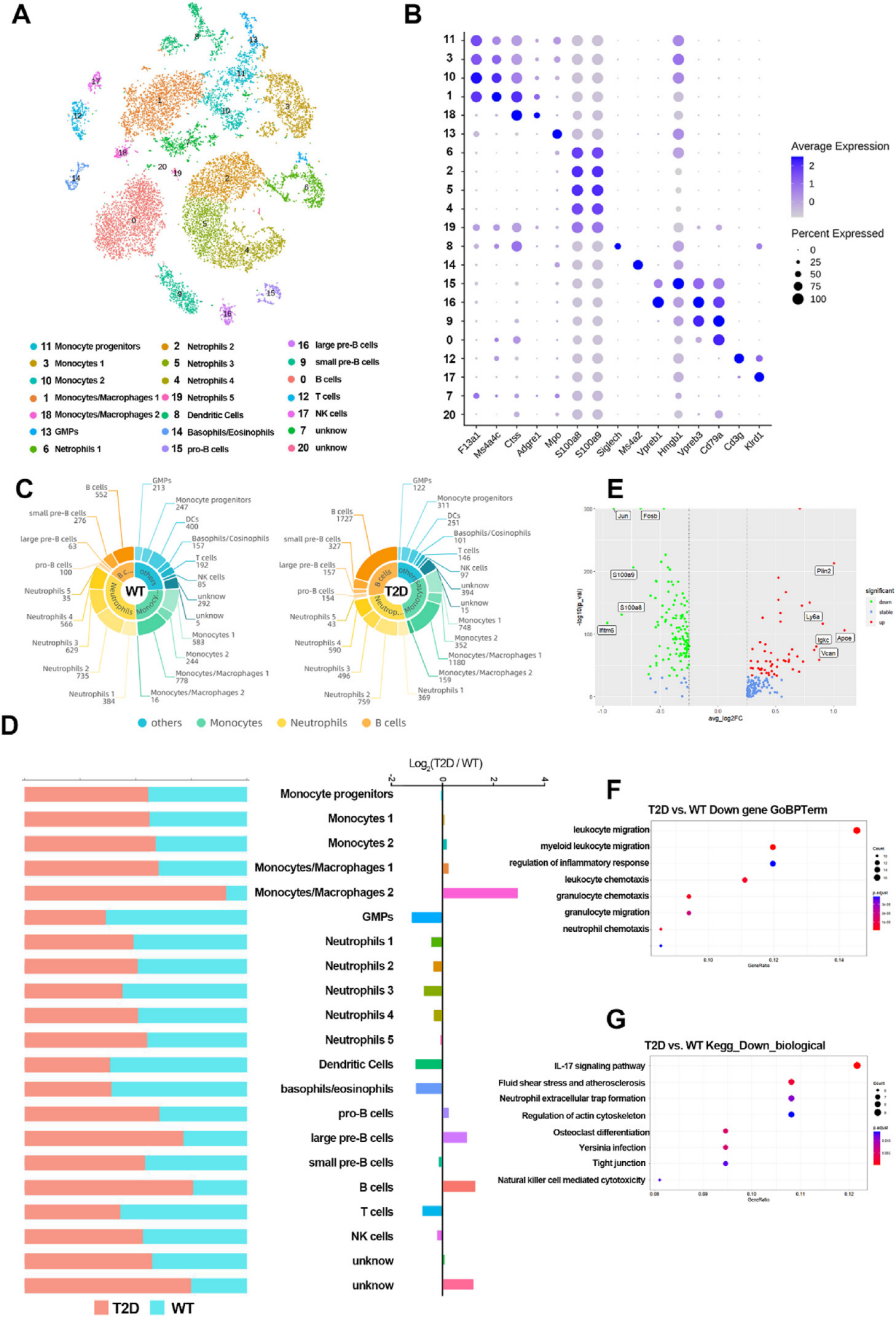
Identification of bone marrow cell populations in WT and T2D mice by scRNA-seq. **(A)***t*-SNE visualization of all single cells colored by cell clusters in the BM. **(B)** The marker genes used to identify cell clusters. Color scale, expression level of marker genes across clusters; Dot size, the percentage of cells expressing the marker genes. **(C)** Double donut plot displaying the cell number of each cluster in the BM of WT and T2D mice. **(D)** Comparison of the cell-type frequencies between the T2D and WT groups. Left: The bar graph showing the percentages of cells from T2D and WT mice assigned to each cell cluster. Right: Log ratio of the average fraction in T2D *vs*. WT. **(E)** Volcano plot showing the top five up- or down-regulated genes in T2D *vs*. WT. **(F)** GO enrichment analysis and **(G)** KEGG pathway analysis of the down-regulated DEGs.

We collected BM samples from 7-month-old T2D and WT mice and employed 10 × Genomics to decipher every individual cell transcriptional profile from the BM cells. After quality control filters, single-cell transcriptomic profiles for 6714 and 8885 BM cells of WT and T2D mice were selected respectively for analysis. The co-analysis visually projected the data onto *t*-SNE (*t*-distributed stochastic neighbour embedding) and classified the BM cells into 21 clusters, according to the transcriptomic diversity (Fig. 1A). As detailed below, we identified the cell identity of each cluster based on the significantly overexpressed marker genes provided by Chiara Baccin^20^ and the CellMarker database.^25^ The cell clusters were annotated to monocyte progenitors (mainly expressing *Prtn3*, *Elane*, *Ly6c2*), granulocyte-monocyte progenitors (mainly expressing *Mpo*, *Elane*), monocytes (mainly expressing *Ccr2*, *Ly6c2*, *Ctss*, *Cx3cr1*, *Itgam*, *Cd68*), monocytes/macrophages (mainly expressing *Adgre1*, *Csf1r*, *Fcgr4*, *Ifngr1*, *Cx3cr1*, *Ccr2*, *Itgam*), neutrophils (mainly expressing *S100a8*, *S100a9*, *Ly6g*, *Anxa1*, *Camp*, *Ngp*, *Itgam*), dendritic cells (mainly expressing *Siglech*), basophils/eosinophils (mainly expressing *Ms4a2*), T cells (mainly expressing *Cd3g*, *Cd3d*, *Cd8b1*), natural killer cells (mainly expressing *Klrd1*), progenitor B cells (mainly expressing *Vpreb1*), small precursor B cells (mainly expressing *Vpreb1*, *Vpreb3*), large precursor B cells (mainly expressing *Vpreb3*, *Cd79a*), and B cells (mainly expressing *Cd74*, *Cd79a*, *Cd79b*, *Ms4a1*, *Ly6d*) (Fig. 1A; B; Fig. S2).

The main cell types in BM cells identified were monocyte, neutrophil, and B lymphocyte, which accounted for more than three-quarters of the total cells in the T2D and WT groups, and contained 4 or 5 different clusters each. In WT mice, monocyte, neutrophil, and B lymphocyte accounted for 24.74%, 35.85%, and 15.13% of the total cells, respectively (Fig. 1C). Compared with the WT group, the proportion of granulocyte-monocyte progenitors, dendritic cells, basophils/eosinophils, T cells, and neutrophil clusters decreased, as well as that of monocytes and most of the B-cell population increased in the T2D group (Fig. 1D). Notably, the monocytes/macrophages 2 (MM2, cluster 18) is distinguished for the much more ratio of cell number between the two groups compared with other clusters (Fig. 1D).

There were 344 DEGs identified between the WT and T2D groups, including 171 up-regulated and 173 down-regulated genes. The top five highly up-regulated and down-regulated DEGs are shown in Figure 1E. Gene ontology (GO) enrichment analysis and kyoto encyclopedia of genes and genomes (KEGG) pathway analysis were performed on the up-regulated and down-regulated DEGs, respectively. The most highly enriched biological process terms among the up-regulated DEGs were the response to B cell activation, mononuclear cell differentiation, and activation of immune response (Fig. S3A). Conversely, the most enriched biological process terms among the down-regulated DEGs were leukocyte migration, myeloid leukocyte migration, regulation of inflammatory response, and leukocyte chemotaxis (Fig. 1F). In addition, the most enriched pathways among the up-regulated DEGs were antigen processing and presentation, B cell receptor signaling pathway, and hematopoietic cell lineage (Fig. S3B). In contrast, the most enriched pathways among the down-regulated DEGs were those associated with the interleukin (IL)-17 signaling pathway, regulation of actin cytoskeleton, and osteoclast differentiation (Fig. 1G).

### Heterogeneity of neutrophils in the bone marrow of T2D versus WT mice

**Figure 2:**
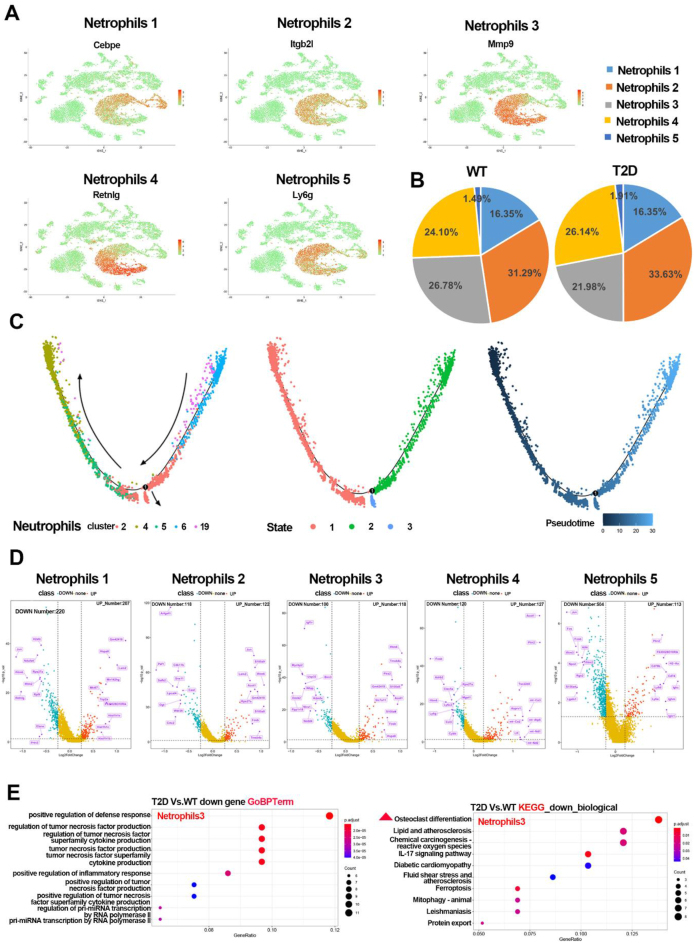
The heterogeneity characteristics of bone marrow neutrophils in T2D mice. **(A)** The cell identity of neutrophil subsets among all cells based on the significantly overexpressed marker genes. **(B)** The proportion of five subsets in neutrophils in the T2D and WT groups. **(C)** Pseudotime analysis of five neutrophil subsets. **(D)** Volcano plot showing the top ten up- or down-regulated genes for five neutrophil subsets. **(E)** GO (left) and KEGG (right) enrichment analysis of down-regulated DEGs in neutrophils 3.

To explore the heterogeneity of neutrophils, we analyzed the characteristics of the neutrophil subsets. The results showed that the neutrophils could be subdivided into five subsets (Fig. 2A): neutrophils 1 (mainly expressing *Fcnb*, *Cebpe*), neutrophils 2 (mainly expressing *Cebpe*, *Itgb2l*, *Ly6g* and *Syne1*), neutrophils 3 (mainly expressing *Mmp8*, *Mmp9*, and *Syne1*), neutrophils 4 (mainly expressing *Cxcr2*, *Mmp9*, *Mxd1*, and *retnlg*), and neutrophils 5 (mainly expressing *Ly6g*). While the proportion of neutrophil/BM cells in the T2D group decreased to 26.56%, compared with that in the WT group (35.85%), the 5 neutrophil subsets were all decreased in the T2D group, among which, neutrophil 3 (cluster 5) had the largest percentage decrease, to be 0.61 times of WT (Fig. 1D). Similarly, when comparing the five subsets among neutrophils between the WT and T2D group, neutrophil 3 was decreased the most (Fig. 2B). To delineate the differentiating orientation among the clusters, we performed pseudotime analysis of the trajectory and status of the neutrophils (cluster 2, 4, 5, 6, and 19) with Monocle 2. The trajectory of the five clusters did not show a clear branched differentiation process or state from progenitor cells to mature cells, while according to the cell annotation, we inferred that cluster 19 (neutrophils 5) and 6 (neutrophils 1) might be upstream of other clusters, while cluster 5 (neutrophils 3) and cluster 4 (neutrophils 4) the downstream (Fig. 2C). Among all DEGs in the five neutrophil subsets, *H2afz*, *Arfgef1*, *Igf1r*, *Acot1*, and *Jun* were DEGs with the most significant changes in proportion (Fig. 2D). Furthermore, GO enrichment analysis (Fig. S4A) and KEGG pathway analysis were performed for the down-regulated DEGs in neutrophil subsets (Fig. S4B). The results showed that most of the biological processes enriched among the DEGs in neutrophil 3 were related to the positive regulation of defense response, regulation of tumor necrosis factor product, positive regulation of inflammatory response, and regulation of pri-miRNA transcription by RNA polymerase II (Fig. 2E). KEGG pathway analysis indicated that osteoclast differentiation, *IL-17* signaling pathway, and ferroptosis were the most highly enriched pathways among the DEGs (Fig. 2E).

### Heterogeneity of B lymphocytes in the bone marrow of T2D versus WT mice

**Figure 3:**
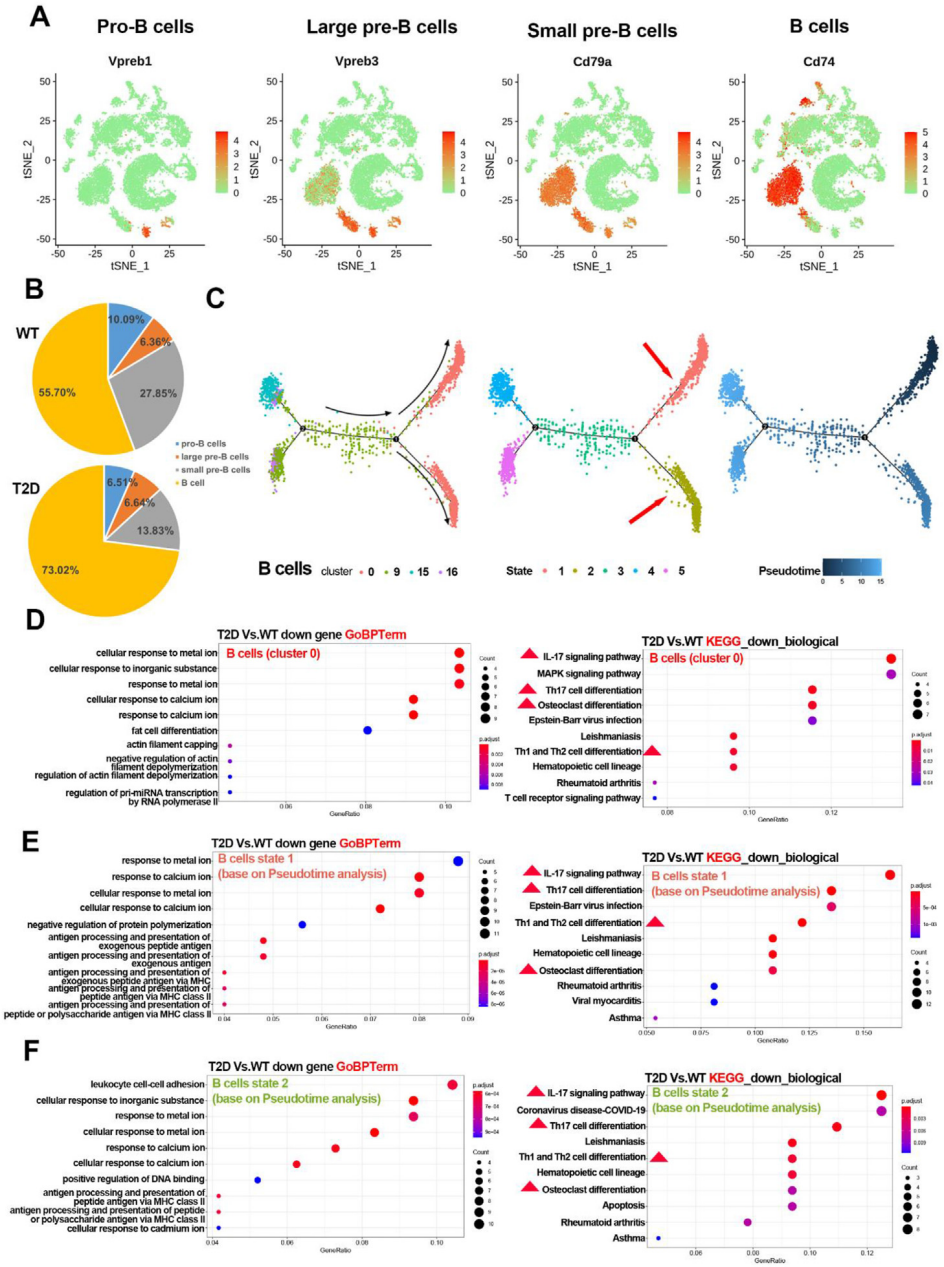
The characteristics of B lymphocytes in T2D mice. **(A)** The cell identity of B lymphocytes subsets among all cells based on the significantly overexpressed marker genes. **(B)** The proportion of B lymphocyte subsets in the T2D and WT groups. **(C)** Pseudotime analysis of B lymphocytes. **(D)** GO (left) and KEGG (right) analysis of down-regulated DEGs of B cells. **(E)** GO (left) and KEGG (right) analysis of down-regulated DEGs of B cells at state 1 (based on Pseudotime analysis). **(F)** GO (left) and KEGG (right) analysis of down-regulated DEGs of B cells at state 2 (based on Pseudotime analysis).

We next examined the characteristics and differentiation of B lymphocytes. The B lymphocytes could be subdivided into four subsets: progenitor B cells (*Vpreb1*), lagre precursor B cells (*Vpreb3*, *Cd79a*), small precursor B cells (*Vpreb1*, *Vpreb3*, *Cd79a*), and B cells (*Cd74*, *Cd79a*, *Cd79b*, *Ms4a1*, *Ly6d*) (Fig. 3A). While the proportion of B lymphocyte/BM cells in the T2D group increased to 27.83%, compared with that in the WT group (15.13%), B cells (cluster 0) had the largest percentage increase among the four subsets, to be 2.41 times of WT (Fig. 1D), which was similar when comparing the four subsets among B lymphocytes between the WT and T2D groups (Fig. 3B). Pseudotime analysis of B lymphocytes (cluster 0, 9, 15, and 16) showed that cluster 15 seemed to be the original source, which was consistent with the cell annotation (progenitor B cells), while cluster 0 were at the most downstream of peusodotime analysis, and might be the mature B cells (Fig. 3C). GO enrichment analysis and KEGG pathway analysis were performed for the down-regulated DEGs in B cells (cluster 0). GO enrichment analysis showed that most of the enriched biological processes were associated with cellular response to metal ions and cellular response to calcium ions (Fig. 3D). KEGG pathway analysis indicated that DEGs were mainly enriched for *IL-17* signaling pathway, *Th17* cell differentiation, *MAPK* signaling pathway, osteoclast differentiation, and *Th1* and *Th2* cell differentiation (Fig. 3D). Furthermore, GO enrichment analysis and KEGG pathway analysis were performed for the down-regulated DEGs in B cells of the state 1 and state 2 following the peusodotime analysis. GO enrichment analysis showed that states 1 and 2 both had the enriched biological processes of cellular response to calcium ion and antigen processing and presentation of exogenous peptide antigen, while state 2 had leukocyte cell–cell adhesion (Fig. 3E and F). KEGG pathway analysis showed that states 1 and 2 both had enriched pathways for *IL-17* signaling, *Th17* cell differentiation, *Th1* and *Th2* cell differentiation, and osteoclast differentiation, while state 1 had more enriched pathways associated with autoimmune (Fig. 3E, F).

### Heterogeneity of monocytes in the bone marrow of T2D versus WT mice

**Figure 4:**
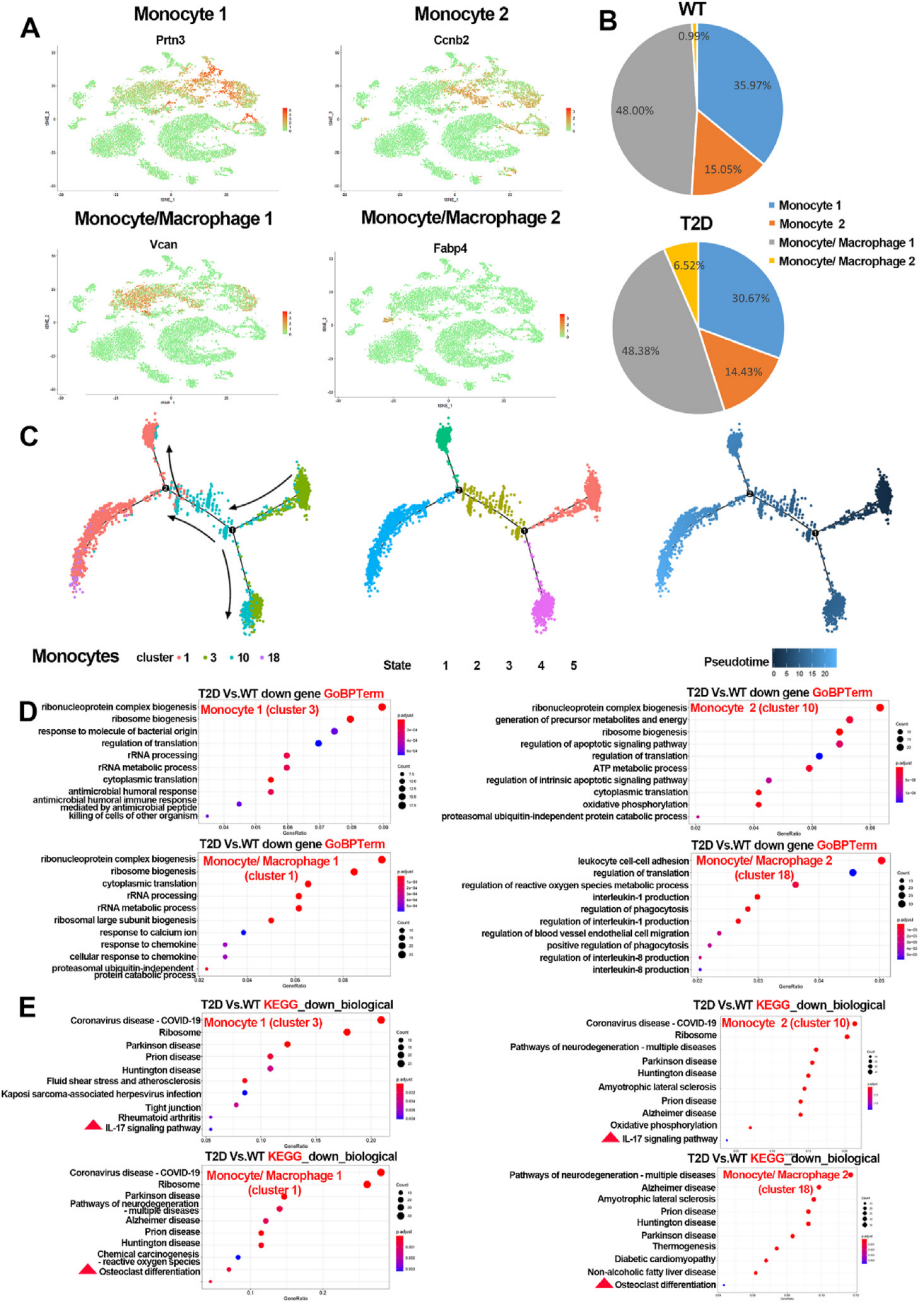
The characteristics of monocyte population in T2D mice. **(A)** The cell identity of monocyte subsets among all BM cells based on the significantly overexpressed marker genes. **(B)** The proportion of monocyte subsets in the T2D and WT groups. **(C)** Pseudotime analysis of monocyte subsets. **(D)** GO analysis of down-regulated DEGs of each monocyte subset. **(E)** KEGG analysis of down-regulated DEGs of each monocyte subset.

To explore the heterogeneity of monocytes, we analyzed the characteristics of the monocyte subsets. The results showed that the monocytes could be subdivided into four subsets (Fig. 4A): monocytes 1 (mainly expressing *Mki67*, *Prtn3*, *Stmn1*, *Top2a*), monocytes 2 (mainly expressing *Ccnb2*, *Cenpa*), monocytes/macrophages 1 (MM1, mainly expressing *Ccr2*, *Fn1*, *LrP1*, *Vcan*, *Wfdc17*), and monocytes/macrophages 2 (MM2, mainly expressing *Ace*, *Adgr4*, *Cd36*, *Fabp4*). While the proportion of monocyte/BM cells in the T2D group was slightly increased to 28.70%, compared with that in the WT group (24.74%), MM2 (cluster 18) had the largest percentage increase among the four subsets, to be 7.66 times of WT (Fig. 1D), which was similar when comparing the four subsets among monocytes between the WT and T2D groups (Fig. 4B). The pseudotime analysis suggested that monocytes 1 (cluster 3) seemed to be the original source of all monocytes and macrophages (cluster 1, 3, 10, and 18), while monocytes 2 (cluster 10) to be the intermediary between monocytes 1 (cluster 3) and monocytes/macrophages (cluster 1 and 18) (Fig. 4C). Furthermore, GO enrichment analysis and KEGG pathway analysis were performed for the DEGs in monocyte subsets. The GO biological processes of several cytokine/chemokine production and response to cytokines in the monocyte population were enriched in the T2D group, such as *IL-12*, *IL-1*, and *IL-6* (Table S1), suggesting that the monocyte population may play a role in the regulation of BM microenvironment in T2D. The analysis of down-regulated DEGs showed that most of the GO biological processes enriched in monocytes 1, monocytes 2, and MM1 (cluster 1) were related to the ribonucleoprotein complex biogenesis, while those in MM2 (cluster 18) were related to the leukocyte cell–cell adhesion, regulation of translation, regulation of reactive oxygen species metabolic process, *IL-1* production, and regulation of phagocytosis (Fig. 4D). KEGG pathway analysis showed that ribosome and *IL-17* signaling pathway were enriched among the DEGs in both monocytes 1 and monocytes 2, while osteoclast differentiation was enriched in both MM1 and MM2 (Fig. 4E).

### Decreased osteoclast differentiation ability of BM cells under the T2D condition

Above KEGG analysis of the total DEGs and DEGs of the most changed clusters, such as B cell (cluster 0), neutrophil 3, and monocyte clusters, all enriched for osteoclast differentiation and/or *IL-17* signaling pathway. To further dissect the transcriptomic features of BM cells in T2D mice related to osteoclast differentiation, we investigated the functions of the DEGs. The top ten up-regulated and down-regulated genes, according to *P* < 0.05 and |log2FC*|* > 0.25 for each cluster, were listed in the volcano plot (Fig. S5).

**Figure 5:**
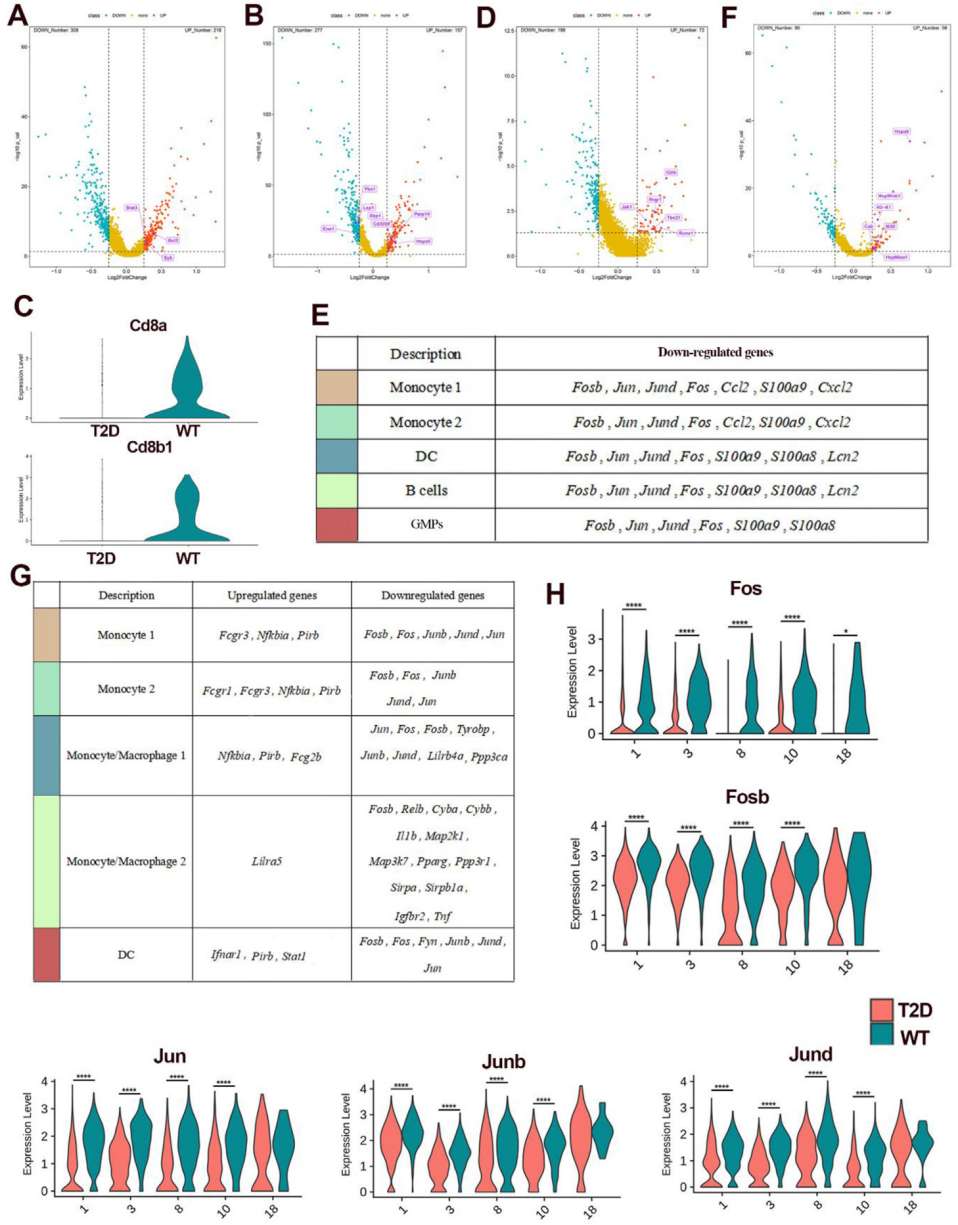
Osteoclast differentiation ability of bone marrow cells in T2D mice. **(A)** The volcano plot showing the up-regulated genes that promote *IL-10* production in monocytes 2 of T2D mice. **(B)** The volcano plot showing the up-regulated genes that promote *IL-4* responses and inhibit the response to *IL-7* in MM1 of T2D mice. **(C)** The violin plots showing the expression levels of *Cd8a* and *Cd8b1* expressed specifically in T cells between WT and T2D mice. **(D)** The volcano plot showing the up-regulated genes that inhibit *Th17* cell differentiation in T cells of T2D mice. **(E)** Down-regulated genes of the *IL-17* signaling pathway in multiple cell types of T2D mice. **(F)** The volcano plot showing the up-regulated genes associated with antigen processing and presentation pathways in B cells of T2D mice. **(G)** Osteoclast differentiation inhibition-associated DEGs in BM monocyte and dendritic cells (DC) populations in T2D *vs*. WT. **(H)** The violin plots showing the expression of *Fos*, *Fosb*, *Jun*, *Junb*, and *Jund* in each cluster of monocyte and DCs populations (1, MM1; 3, monocytes 1; 8, DCs; 10, monocytes 2; 18, MM2) between T2D and WT mice. ^∗^*P* < 0.05, ^∗∗∗∗^*P* < 0.0001.

Cytokines play a key role in regulating osteoclast differentiation. The DEGs analysis showed that several osteoclast differentiation-related cytokine signals in different clusters were obviously affected in the BM immune microenvironment of T2D mice. In monocytes 2, genes that promote *IL-10* production (*Bcl3*, *Stat3*, *Syk*) were up-regulated (Fig. 5A). In MM1, genes that promote *IL-4* response (*Cd300lf*, *Xbp1*, *Hspa5*, *Parp14*) were up-regulated, while genes that inhibit *IL-7* response (*Lsp1*, *Ybx1*, *Eno1*) were down-regulated (Fig. 5B). In T cells, the *Cd8* coding genes (*Cd8a* and *Cd8b1*) were down-regulated dramatically in the T2D group (Fig. 5C), while those genes that inhibited *Th17* cell differentiation, including *Tbx21*, *Il2rb*, *Runx1*, *Ifngr1*, and *Jak1,* were increased in the T2D group (Fig. 5D; Fig. S6). Meanwhile, the *IL-17* signaling pathway of most immune cells in the BM was generally down-regulated under T2D conditions, which was reflected in the down-regulation of transcription factors and expression products of this pathway (Fig. 5E; Fig. S5). Moreover, the pathway of antigen processing and presentation (*Hspa8*, *Hsp90ab1*, *H2–K1*, *Calr*, *Ifi30*, *Hsp90aa1*) was enriched by the up-regulated genes in the B cells of T2D mice, which is associated with B cell activation (Fig. 5F; Fig. S7).

It is generally believed that monocytes, macrophages, and dendritic cells in the BM can develop into osteoclasts. To further investigate the trend of osteoclast differentiation in the T2D group, we analyzed the DEGs associated with osteoclastic differentiation of monocyte and dendritic cell populations in the BM. The results showed that the genes related to osteoclast differentiation inhibition were mostly located downstream of the signaling pathway, such as the down-regulated expression of the genes encoding the protein subunit that constitutes the transcription factor *AP-1* (*Fosb*, *Fos*, *Jun*, *Junb*, *Jund*) and the up-regulated expression of the gene encoding IκBα (*Nfkbia*), which has the function of inhibiting the NF-κB transcription factor (Fig. 5G). In addition, gene enrichment analysis showed that the inhibited signaling pathway of osteoclast differentiation in different cell populations was mainly concentrated in the *AP-1* pathway, and the genes related to *AP-1* were generally down-regulated in the monocyte and dendritic cell populations under the T2D condition (Fig. 5H).

### A unique monocyte/macrophage cluster in the BM of T2D mice

**Figure 6:**
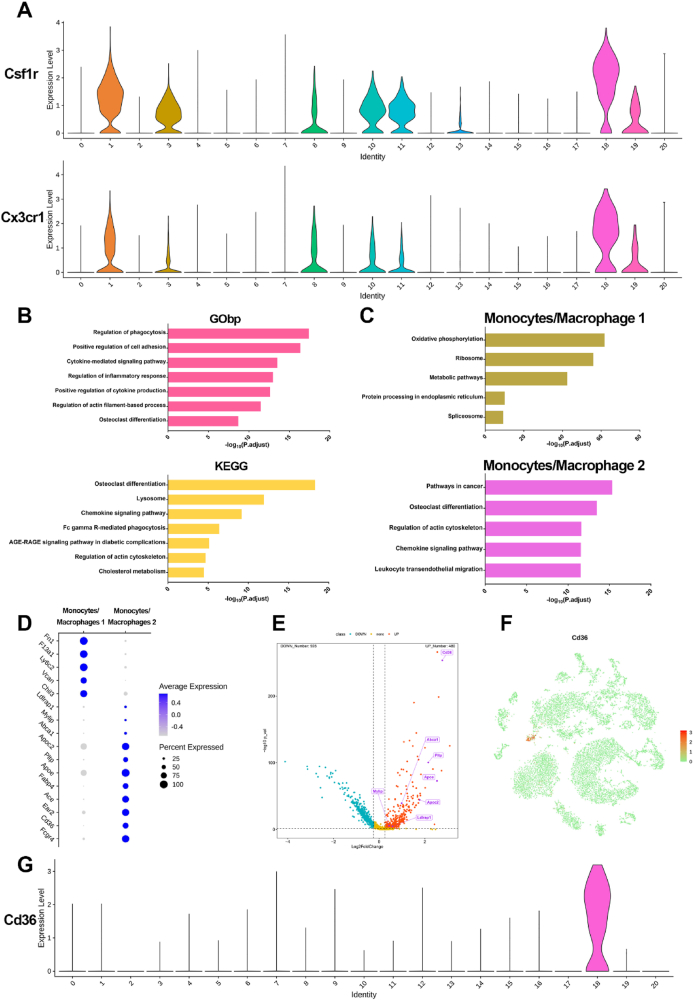
Characteristics of a unique cluster, MM2. **(A)** The violin plots showing the expression of selected cell-type-specific markers (*Csf1r*, *Cx3cr1*) in MM2 (cluster 18) and other clusters. **(B)** The top seven enriched pathways of all marker genes (*P* < 0.05, log2FC > 0.25) for MM2 achieved by GO and KEGG enrichment analysis. **(C)** Top five KEGG pathways in MM1 and MM2. **(D)** Dot plots of the enriched DEGs in MM1 and MM2. **(E)** Volcano plot of all gene expression in MM1 versus MM2. Cholesterol metabolism-related genes highly expressed in MM2 are in purple. **(F)** The *t*-SNE plots showing the expression of *Cd36*. **(G)** The violin plots showing the expression of *Cd36* for each cell cluster.

From the above results, we observed that the proportion of MM2 (cluster 18) in T2D mice was obviously higher than that in WT mice (Fig. 1D), and this cluster even hardly exists in WT mice (Fig. 1C), suggesting that MM2 might be a unique cell cluster in T2D. MM2 had the characteristics of monocytes and expressed some gene markers of macrophages (*Csf1r*, *Cx3cr1*) (Fig. 6A). To testify the validity of the cell annotation of MM2, we analyzed the potential biological processes and pathways of all candidate marker genes for MM2. We carried out GO and KEGG analysis of the candidate marker genes, and found enrichment of osteoclast differentiation, lysosome, and regulation of phagocytosis, *etc*. in MM2 (Fig. 6B). These functions and signaling pathways illustrated that MM2 does exhibit macrophage-related functions and has a close relationship with osteoclasts. Interestingly, we found that MM2 exhibited increased expression of *Thbd*, *Cybb*, *Stat3*, and *Prkcd*, which were associated with the *AGE-RAGE* signaling pathway in diabetic complications (Fig. 6B). In addition, pseudotime trajectory analysis using Monocle 2 showed that MM2 was located at the end of the MM1 branch, indicating that it may be derived from MM1 (Fig. 4C). To comprehensively dissect the uniqueness of MM2, we focused on MM1 and MM2 clusters and explored the underlying differences between them. KEGG and DEGs analyses indicated that basal metabolism-related genes were highly enriched in MM1, such as enrichment of ribosome and spliceosome, suggesting cell growth and proliferation due to protein synthesis or positive regulation of cell differentiation (Fig. 6C, D); osteoclast differentiation and chemokine signaling pathway genes were highly enriched in MM2 (Fig. 6C, D), and cholesterol metabolism-related genes such as *Apoe*, *Pltp*, *Cd36*, and *Apoc2* showed highly enriched expression (Fig. 6D, E). These results suggested that MM2 may be a unique state of monocytes/macrophages in T2D mice. To determine whether MM2 indeed exists among the BM cells, we identified *Cd36* as a cell surface marker gene of this cluster, which was expressed in more than 70% of MM2 cells and was significantly up-regulated among other cell clusters (Fig. 6F, G).

### Identification of *Cd36*^*+*^ monocytes/macrophages in the BM of T2D mice

**Figure 7:**
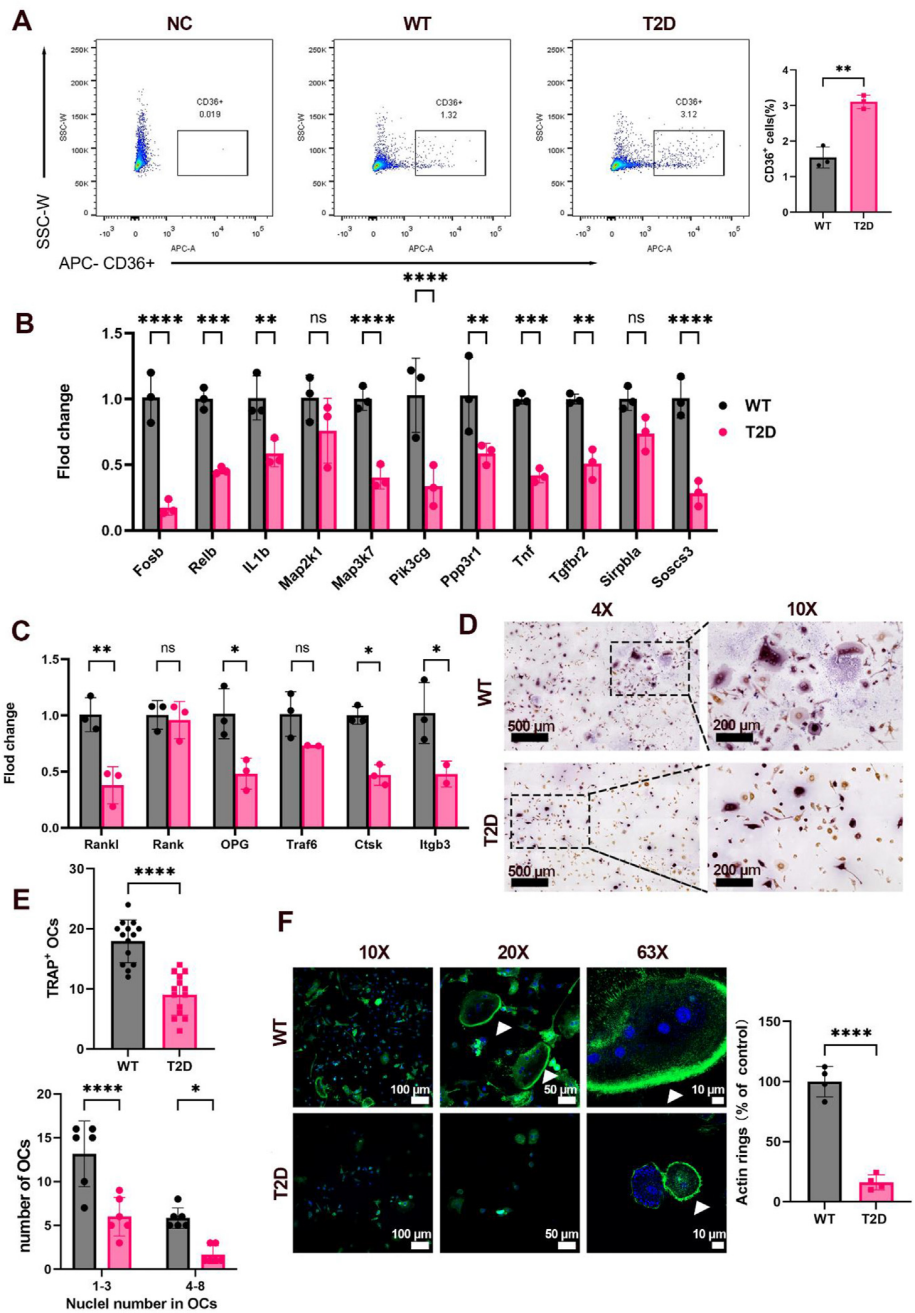
Identification of *Cd36*^*+*^ monocytes/macrophages in the bone marrow of T2D mice. **(A)** Flow cytometry analysis of *Cd36*^*+*^ cells in the BM from 7-month-old WT and T2D mice. NC, negative control. **(B, C)** qPCR analysis of the relative mRNA expression of scRNA-seq identified and reported osteoclast differentiation-associated genes in sorted *Cd36*^*+*^ BM cells. **(D)** Representative images of TRAP staining. **(E)** Number of TRAP^+^ stained osteoclasts (OCs) and nuclei number in OCs. **(F)** Actin ring formation analyzed by phalloidin staining (green). DAPI, blue. White arrow, actin ring. *n* = 3–6 per group. Results were expressed as mean ± standard deviation. ^∗^*P* < 0.05, ^∗∗^*P* < 0.01, ^∗∗∗^*P* < 0.001, ^∗∗∗∗^*P* < 0.0001.

To verify the existence of MM2 in T2D mice, we collected the BM cells from WT and T2D mice and used a *Cd36*^*+*^ antibody to label MM2 with flow cytometry. The results confirmed that the frequency of *Cd36*^*+*^ cells in the BM of T2D mice was significantly increased (Fig. 7A). Then we sorted *Cd36*^*+*^ cells with flow cytometry and performed qPCR detection to verify the expression changes of transcripts associated with osteoclast differentiation identified by scRNA-seq in T2D mice (Fig. 7B). The results showed that the expression levels of *Fosb*, *Relb*, *IL1b*, *Map3k7*, *Pik3cg*, *Ppp3r1*, *Tnf*, *Tgfbr2*, and *Soscs3* were significantly decreased in the *Cd36*^*+*^ cells of T2D mice. Among them, the key downstream genes that control osteoclast differentiation, such as *Fosb* (encoding a component of *AP-1*) and *Relb* (encoding *Relb* of the *NFκB* family), were significantly down-regulated. Furthermore, qPCR analysis of some known osteoclast differentiation-related genes showed that *RANKL*, *OPG*, *Ctsk*, and *Itgb3* were significantly down-regulated, compared with the WT group (Fig. 7C). In bone mononuclear macrophages obtained from T2D and WT mice, TRAP^+^ osteoclasts and nuclei number in osteoclasts (Fig. 7D, E), and the number of intact actin rings (Fig. 7F) all significantly decreased in T2D mice, compared with those in WT mice. Overall, we believe that the *Cd36*^*+*^ cell population in the T2D environment reduces the differentiation of monocytes/macrophages to osteoclasts.

## Discussion

Previous studies have emphasized that there is extensive communication and connection between the skeletal system and the immune system, especially within the BM microenvironment.^26^, ^27^, ^28^, ^29^ The significant impact of the bone immune system on bone health and disease has greatly changed the approach we use to treat bone pathologies, conferring highly promising therapeutic opportunities.^9^ Given that diabetes adversely affects bone health and many studies have focused on abnormal bone metabolism in the diabetic setting,^6^^,^^7^^,^^30^, ^31^, ^32^ the role of osteoimmunology within the BM microenvironment under T2D conditions, however, has not been well studied. Therefore, understanding the basic cellular and molecular mechanisms underlying osteoimmunology is essential for T2D-related bone disease. In the present study, we delineated the single-cell transcriptome of BM immune cells from both wild-type and T2D mice, which provides the first detailed scRNA-seq description of the changes in osteoimmunology and bone metabolism, and T2D-induced gene alterations in the BM microenvironment.

We conducted a scRNA-seq analysis to understand the varieties of BM cells under T2D conditions for the first time. The results presented an unbiased clustering of all cells. Furthermore, we examined the differentiation of cell subsets in the BM microenvironment in the T2D condition. We divided mouse BM cells into 12 main cell types with 21 clusters, mainly including granulocyte-monocyte progenitors, monocyte progenitors, monocytes, monocytes/macrophages, neutrophils, eosinophils, basophils, B cells, T cells, and natural killer cells; and monocytes, neutrophils, and B cells accounted for more than three-quarters of the total cells. Recently, Zhong et al reported the results of scRNA-seq in streptozotocin-induced type 1 diabetes mice and revealed an inverse relationship between the proportion of neutrophils/B lymphocytes in BM and osteopenia.^33^ Although the decrease of bone mineral density in the type 1 diabetes population was reported in almost all the epidemiological surveys, the bone mineral density in the T2D population had not been reported to decrease, or even increase in many studies.^6^^,^^34^^,^^35^ Therefore, the underlying mechanism of bone fragility and bone disease in T2D looks different from that in type 1 diabetes. In fact, in the present study, we found a totally different profile of BM cells in T2D mice compared with that reported in type 1 diabetes mice.^33^ We found that compared with the WT group, the frequency of granulocyte-monocyte progenitors, dendritic cells, neutrophils, eosinophils/basophils, and T cells all decreased in T2D mice, while that of B cells and large precursor B cells increased significantly. Notably, the most significant difference between the two groups was the proportion of MM2, which indicated that these kinds of cells may be more vulnerable to T2D conditions than other BM cell types. The KEGG enrichment analysis of total DEGs and DEGs of the most changed clusters indicated that osteoclastic differentiation and/or *IL-17* signaling pathway are generally affected in T2D mice. Then we explored the possible function changes of the bone immune cells and their interaction and correlation with bone metabolism in the BM microenvironment under T2D conditions.

Numerous studies have found that hyperglycemia can lead to immunity dysfunction in patients.^36^ It was reported that there is an abundant increase in resident macrophages in the tissues of patients with T2D^37^; however, these macrophages usually show reduced phagocytosis and abnormal activation.^38^ Our DEGs analysis of scRNA-seq also revealed that the phagocytosis of monocytes/macrophages in the BM of T2D mice was down-regulated in comparison to WT mice (Fig. 4D). On the other hand, substantial evidence suggests that inflammatory and *CD4*^*+*^ T cell differentiation are unbalanced in obese patients with T2D,^39^ which generally suggests that the number of *Th17* cells increases in T2D patients.^40^, ^41^, ^42^, ^43^, ^44^ Santopaolo et al analyzed residual BM cells from patients undergoing hip replacement surgery and found that *CD4*^*+*^ T-cells were more likely to polarize into proinflammatory *Th1* and *Th17* cells in the peripheral blood and adipose tissue of T2D patients, and anti-inflammatory *Th2* cells were relatively reduced.^45^ It was also reported that the frequency of *CD4*^*+*^ T-cells and *CD8*^*+*^ T-cells in BM cells of T2D patients was increased and the expression of the activation marker *CD69* and homing receptor *CCR7* of *CD4*^*+*^ T-cells and *CD8*^*+*^ T-cells was up-regulated.^46^ In the present study, our results revealed very different findings. We found that not only the number of T cells decreased in the BM microenvironment of T2D mice, but also the genes encoding downstream signaling that inhibit differentiation of T cells into *Th17* cells increased, which meant the potential of T cells in BM to develop into *Th17* cells is reduced in mice with T2D. Moreover, as studies on the effects of cytotoxic *CD8*^*+*^ T-cells on bone metabolism are insufficient and remain controversial, we found that the genes encoding *CD8* (*Cd8a*, *Cd8b1*) were significantly down-regulated in T2D mice, suggesting reduced cytotoxic effects of *CD8*^*+*^ T-cells under T2D conditions. We considered that further study focusing on osteoimmunology and bone metabolism within the BM microenvironment under diabetic conditions may contribute to a better understanding of these aforementioned phenomena.

Bone metabolism, also known as bone remodeling, is composed of bone formation and bone resorption, and the osteoblasts and osteoclasts play the key role in the process, respectively. Although the mechanisms of diabetic bone disease have been extensively explored, the majority of research has focused on the osteoblasts,^47^ while the effects of diabetes on osteoclasts remain unclear and controversial.^48^, ^49^, ^50^, ^51^, ^52^, ^53^ Our results showed that although there was less variation in the number of BM monocyte populations between T2D and WT mice, cytokine production involved in the inflammatory response in monocytes/macrophages was significantly altered under T2D conditions. *IL-10* production and *IL-4* response (reported as anti-osteoclastogenic cytokines) were promoted and *IL-7* response (reported as osteoclastogenic cytokines)^26^ was inhibited, indicating that osteoimmune system may regulate bone metabolism through an osteoclast inhibition microenvironment in T2D mice. In the BM microenvironment, *Th17* cells are considered to have the function of promoting osteoclastogenesis,^27^^,^^28^ and the *IL-17* released by *Th17* cells can promote osteoclast differentiation.^54^^,^^55^ As mentioned above, we found that the potential of T cells to differentiate into *Th17* cells was relatively reduced in the BM microenvironment under T2D conditions, and the *IL-17* signaling pathway of most BM immune cells including monocytes, neutrophils, and B cells was generally down-regulated in T2D mice. These findings suggested an osteoclast inhibition microenvironment in the BM of T2D mice. Furthermore, we systematically analyzed the osteoclast differentiation potential of several known osteoclast precursors in the BM under T2D conditions with DEG analysis. We found that many osteoclastic differentiation-related signaling pathways, including *NFκB*, *MAPK*, calcium, and *Jak-STAT*, were inhibited to varying degrees. *AP-1*, a key transcription factor promoting osteoclast differentiation,^56^^,^^57^ was observed significantly down-regulated in these processes. As a critical downstream target of the *RANKL* signaling pathway, the *AP-1* protein plays an important role in regulating osteoclast differentiation by promoting the expression of osteoclast-specific target genes.^57^^,^^58^ The *Fos* family (*v-Fos*, *c-Fos*, *FosB*, *Fra1*, and *Fra2*) and *Jun* family (*v-Jun*, *c-Jun*, *JunB*, and *JunD*) are the main components of the *AP-1* dimer complex in mammals.^59^ Studies have shown that mice lacking the *Fos* gene exhibit a complete loss of osteoclasts.^56^ Our scRNA-seq results showed that the gene expression of the *Fos* family and *Jun* family in the T2D group was down-regulated compared with the WT group, especially *Fosb*, which was also certificated by subsequent biological tests. These findings suggested that the down-regulation of *AP-1*-related gene expression should be the key reason for the decreased osteoclast differentiation in T2D mice, which might compose the mechanism of bone homeostasis imbalance in T2D-related bone disease.

Macrophages and osteoclasts both derive from and compete for differentiation outcomes of monocyte/macrophage-lineage cells. In this study, we observed a specific cell cluster MM2 in T2D mice, which was proved to be the precursor cells with osteoclastic differentiation potential by the DEG analysis. Pseudotime analysis showed that the MM2 cluster was located at the end of the MM1 cluster and enriched the osteoclastic differentiation and chemokine signaling pathway, indicating that the MM2 cluster may be a detention or intermediate state between monocytes/macrophages and mature osteoclasts. In addition, MM2 might be a unique cell cluster that can be easily observed under T2D conditions because the percentage of MM2 in the WT group was very rare. The characteristic marker of the MM2 cluster, *Cd36*, was highly expressed. We purified the MM2 subset from mouse BM using a *Cd36* marker and found that compared with the WT group, the frequency of *Cd36*^*+*^ cells in the BM increased significantly in T2D mice by flow cytometry analysis and sorting. The results of TRAP staining and actin ring formation analysis verified decreased osteoclastogenesis and osteoclastic differentiation potential of the sorted cells. Further qPCR analysis showed that most of the genes that promote osteoclastic differentiation decreased significantly in T2D mice, and the *Fosb* gene showed the greatest decrease. These results supported the hypothesis that the osteoclast differentiation potential of the MM2 cluster decreased in the T2D setting and *Fosb/AP1* was an important component of the underlying mechanism.

## Conclusions

**Figure 8:**
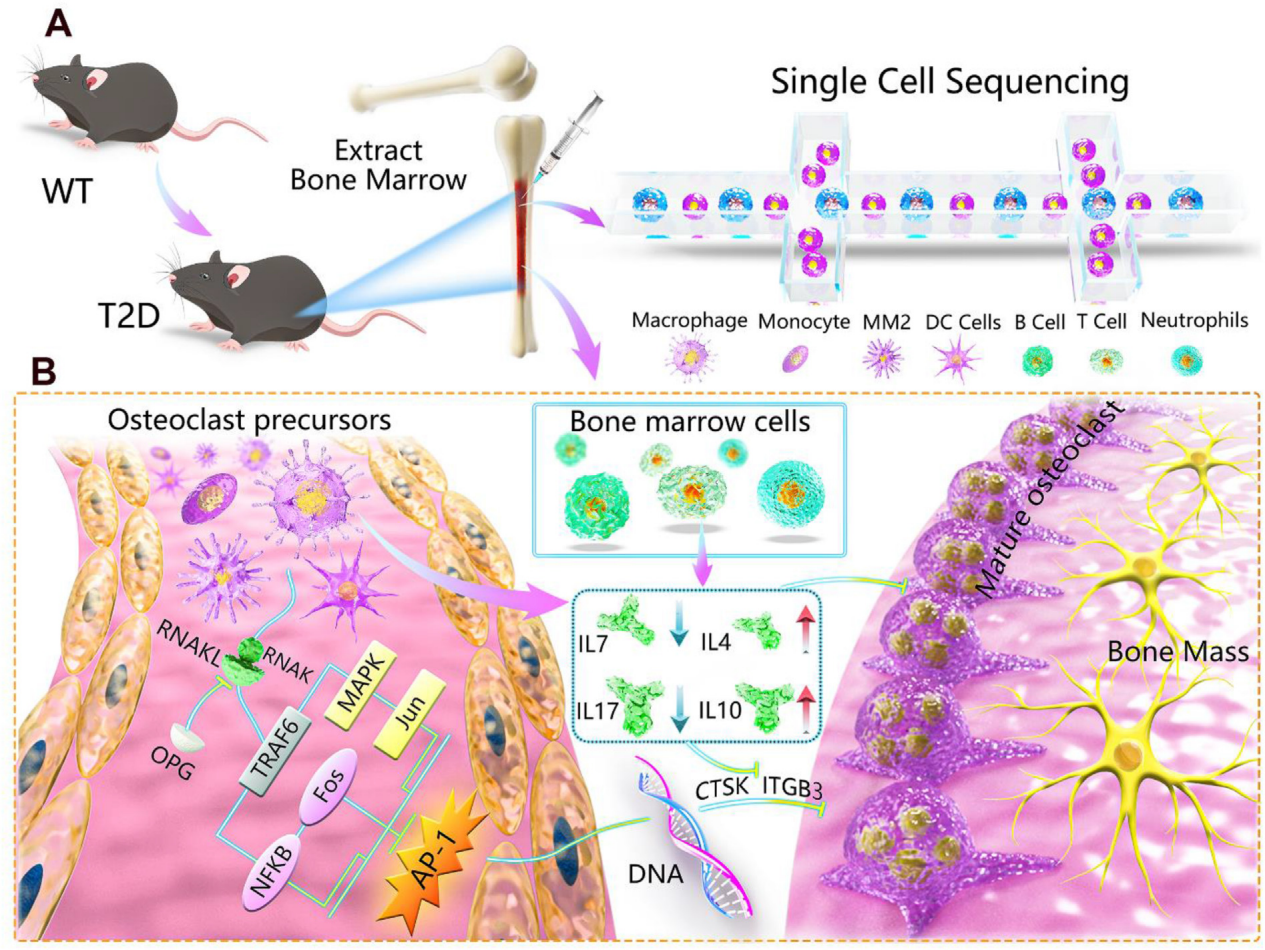
Working model schematic diagram. **(A)** Single-cell sequencing analysis process. **(B)** The mechanism of osteoclast precursor cell differentiation inhibition and the mechanism of bone marrow cells secreting related inflammatory factors to further inhibit osteoclast differentiation.

This study firstly systematically analyzed BM cells in T2D mice by scRNA-seq (Fig. 8A) and explored the T2D-induced cellular and molecular changes in the BM microenvironment (Fig. 8B). From the perspective of osteoimmunology, the detailed global profile of BM cells was delineated at the single-cell level, and the evidence of osteoclast inhibition was further clarified, which including a dysregulated cytokine network leading to decreased osteoclastogenesis and osteoclastic differentiation and identification of *Cd36*^*+*^ cells (MM2) *in vivo* in the T2D environment. The key target *AP-1* was an important transcription factor in the underlying mechanism, laying a foundation for the pathogenesis of diabetes-related bone disease research.

## Author contributions

ZMW, QDH, and HC contributed equally to this work. ZMW designed the overall research. ZMW, QDH, TTC, and KKY performed experiments. ZMW, JHL, QDH, YXM, and JNZ analyzed data. ZMW, HC, and QDH wrote the manuscript. All authors read and edited the manuscript before giving final approval for the version to be published. All authors read and approved the final manuscript. JX, FXW, and LW are responsible for the integrity of the work as a whole.

## Conflict of interests

The authors declare that there are no competing interests.

## Funding

This work was supported by the 10.13039/501100001809National Natural Science Foundation of China (No. 81972045), the Basic Applied Basic Research Foundation of Guangdong Province, China (No. 2022A1515012373), and the research fund from SUSTech Hospital.

## Data availability

The datasets generated during or analyzed during the current study are available from the corresponding author upon reasonable request.
