# Supplementary material for: Single-cell RNA sequencing reveals a distinct profile of bone immune microenvironment and decreased osteoclast differentiation in type 2 diabetic mice
Source: Genes Dis. 2023 Oct 17;11(6):101145. doi: 10.1016/j.gendis.2023.101145 (PMC11399629; doi:10.1016/j.gendis.2023.101145)
Supplement: Multimedia component 1 [file mmc1.docx]

Supporting Information

Single-cell RNA Sequencing Reveals a Distinct Profile of Bone Immune Microenvironment and Decreased Osteoclast Differentiation in Type 2 Diabetic Mice

Zimei Wu, Qiaodan Hou, Heng Chi, Jihong Liu, Yixin Mei, Tingting Chen, Kunkun Yang, Jingna Zheng, Jing Xu ^*^, Fuxin Wei ^**^, Lin Wang ^***^

**Figure. S1**


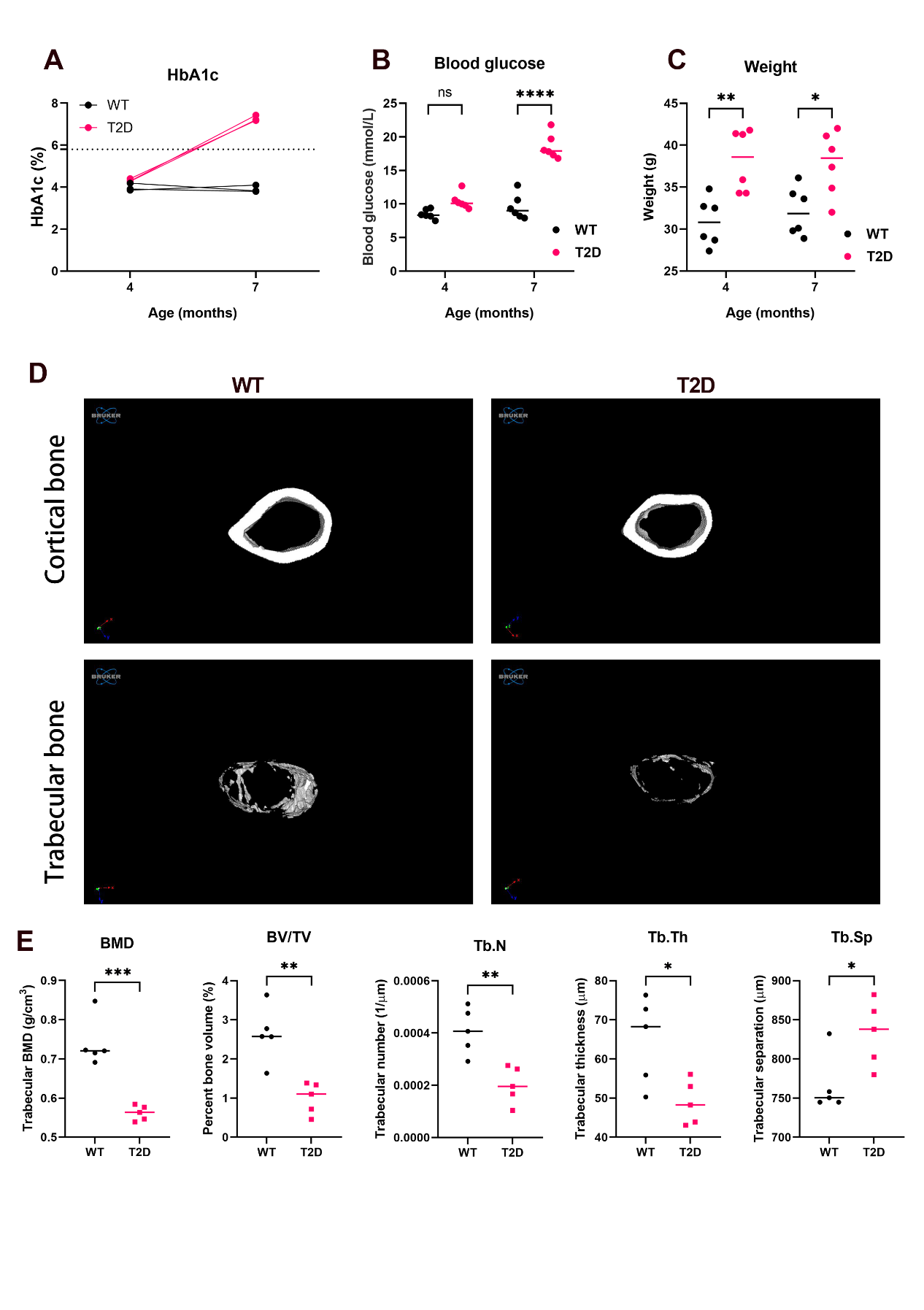


**Figure. S1** T2D mouse model and T2D induced compromised quality of bone mass at seven months.The HbA1C levels (A) and blood glucose levels (B) and fasting body weight(C). Representative images from micro-CT scanning show the cortical (above) and trabecular bone mass (below) in mice: above; cross-section images of the middle diaphysis of tibia. Below; 3-dimensional (3D) reconstruction of the trabecular bone in proximal tibia below (D) Quality parameters of the trabecular bone in proximal tibia metaphysis from micro-CT analysis (E). BMD, BV/TV, trabecular bone volume fraction; Tb.N, trabecular number; Tb.Th, trabecular thickness; Tb.Sp, trabecular separation. n = 5 per group. *p < 0.05, **p < 0.01, ***p < 0.001 and ****p < 0.0001.(A-C) two-way ANOVA followed by Tukey posttest analysis and (E) unpaired t test.

**Figure. S2**


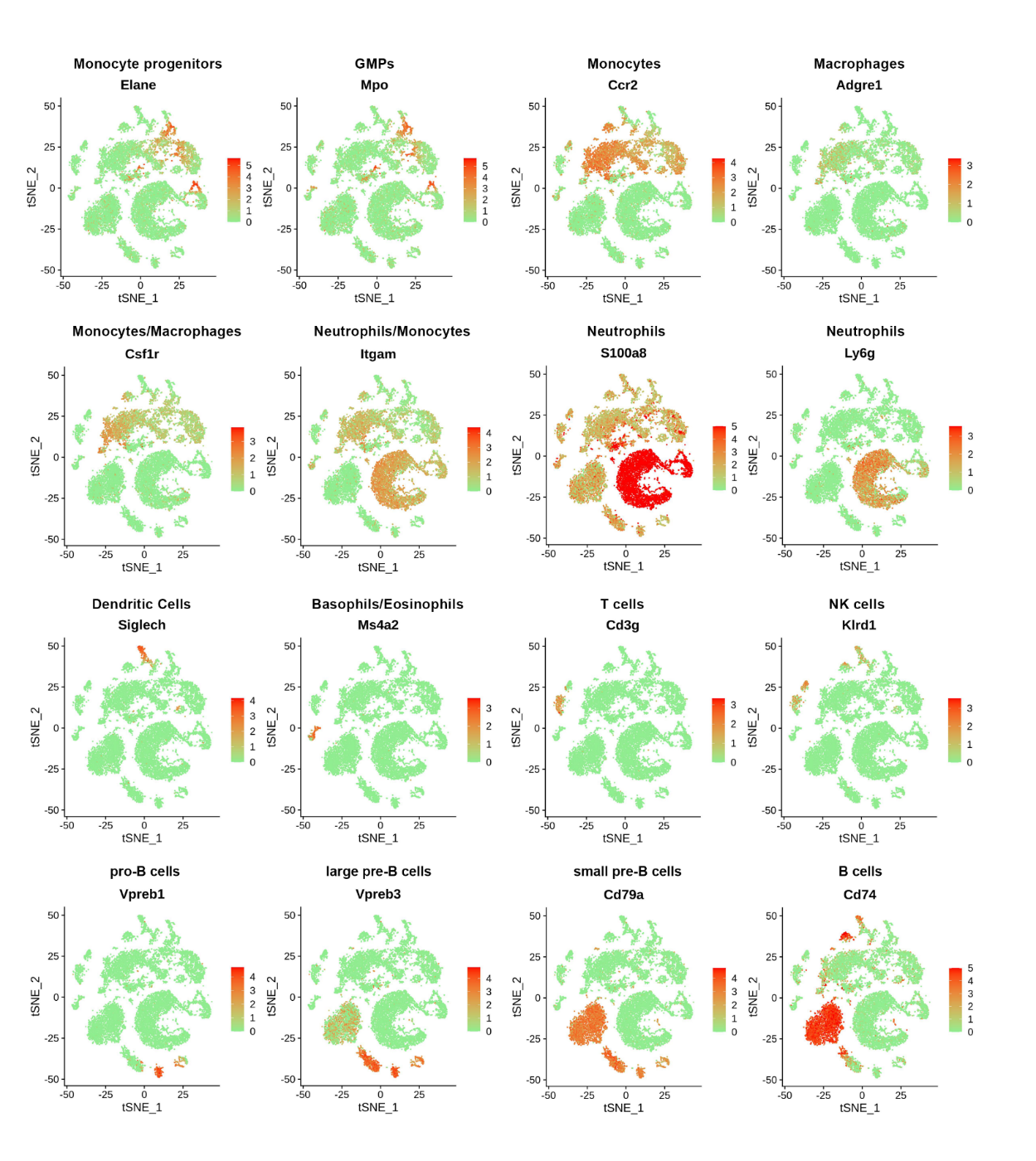


**Figure. S2** The cell identity of each cluster based on the significantly overexpressed marker genes provided by Chiara Baccin and the CellMarker database.

**Figure. S3**


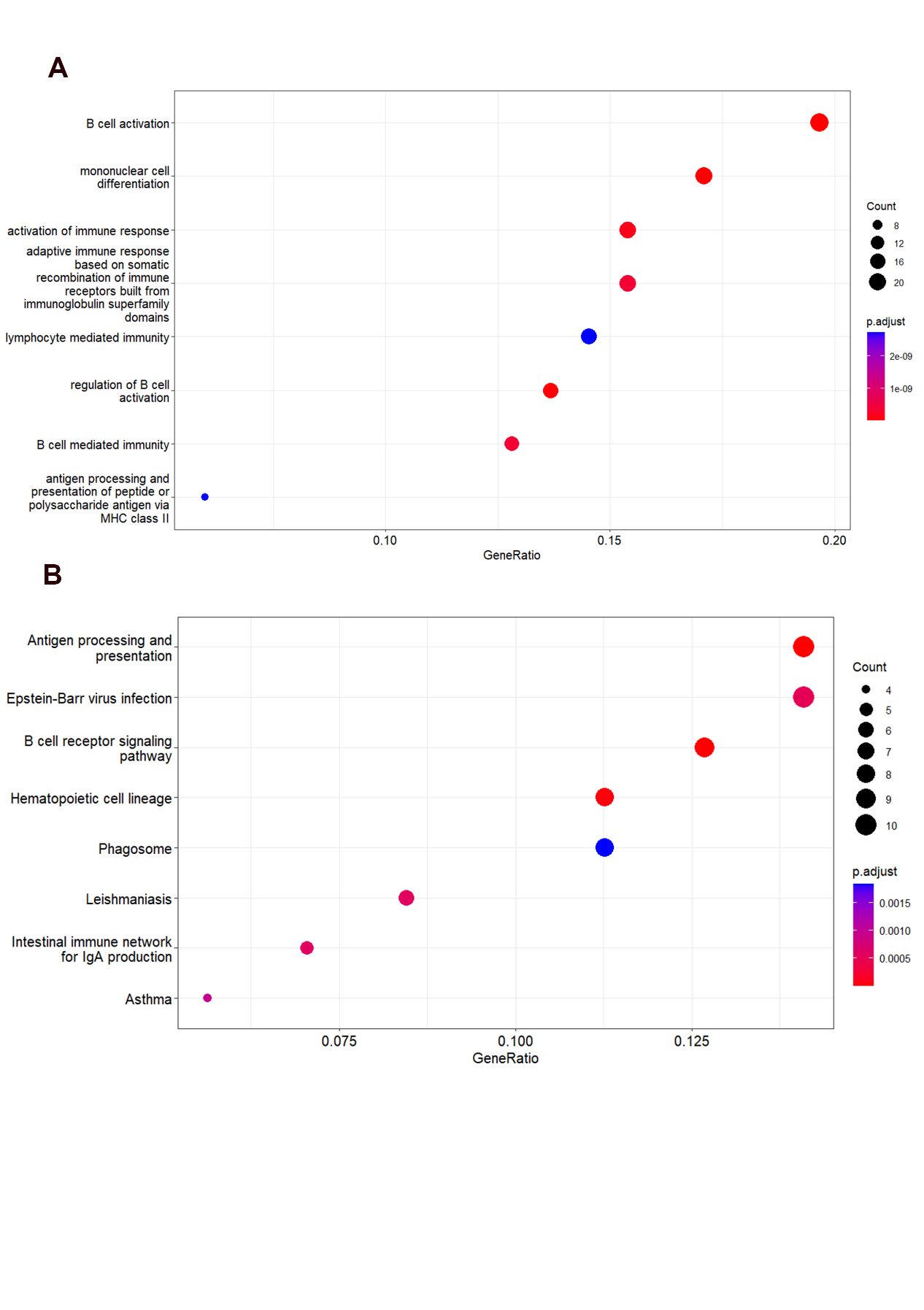


**Figure. S3** GO enrichment analysis **(A)** and KEGG pathway analysis **(B)** of the up-regulated DEGs between WT and T2D mice.

**Figure. S4**

**
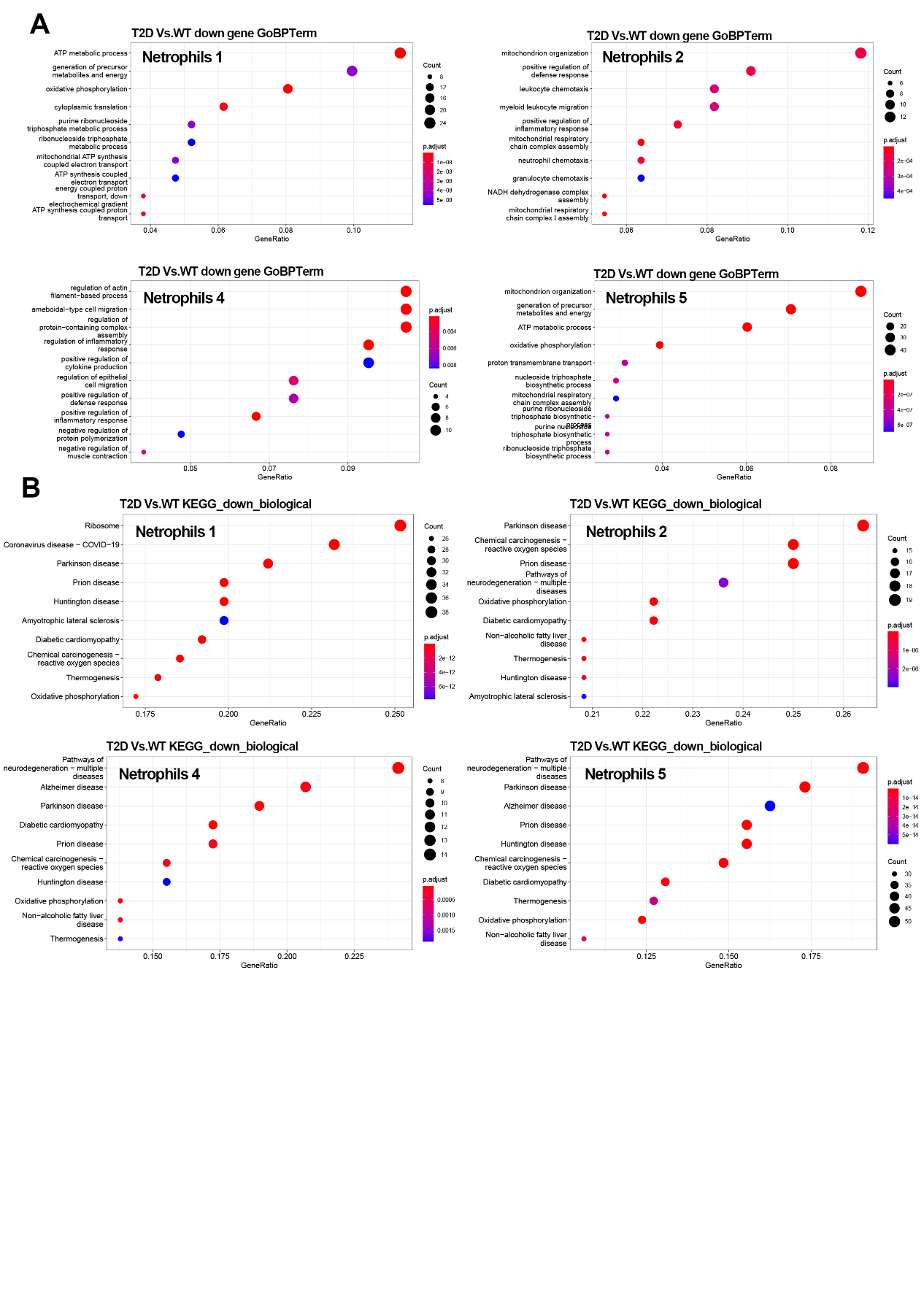
**

**Figure. S4** GO enrichment analysis **(A)** and KEGG pathway analysis **(B)** of the down-regulated DEGs of neutrophil 1, 2, 4, and 5 subsets.

**Figure. S5**


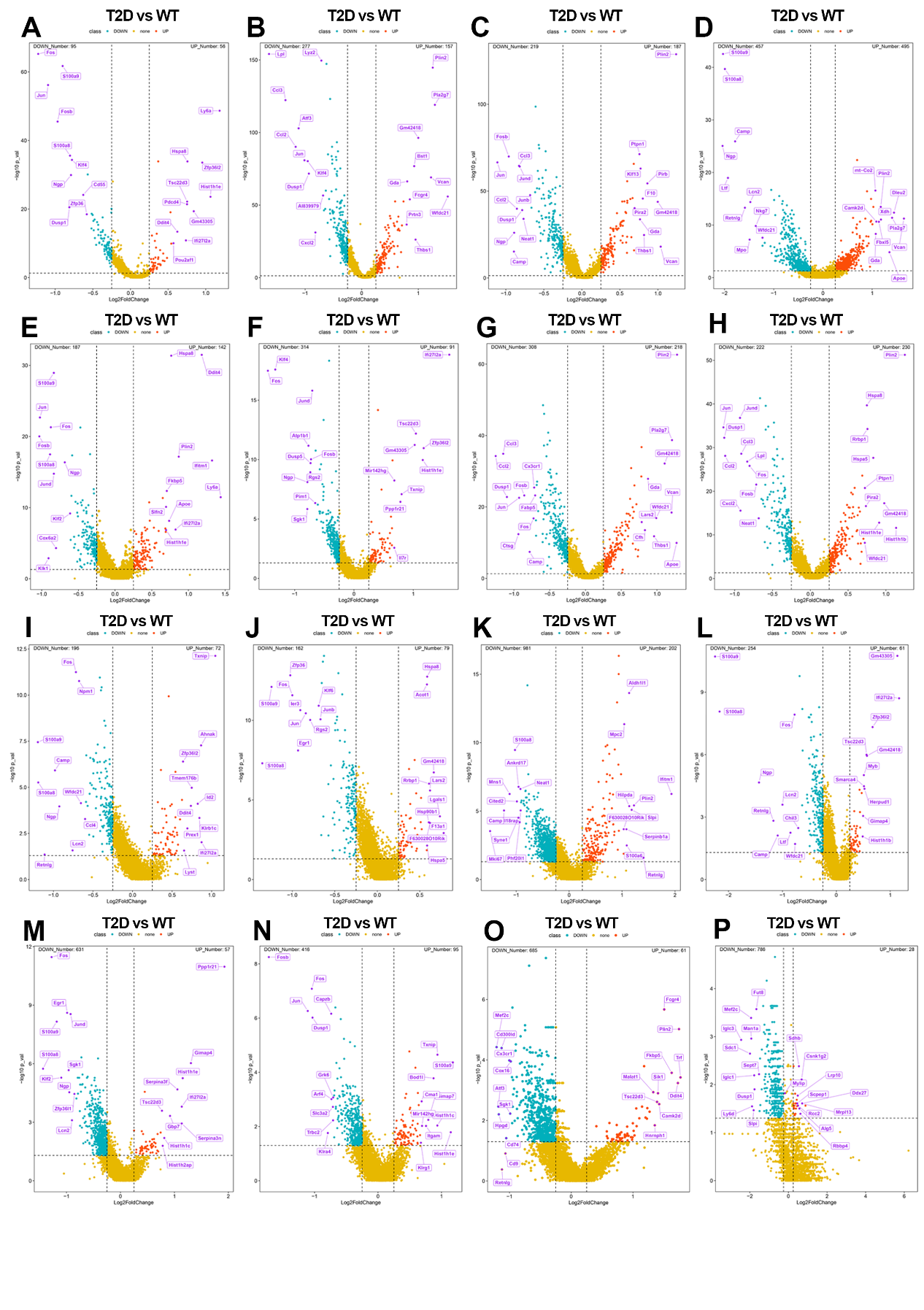


**Figure. S5** The volcano plot showing the top ten up- or down-regulated genes for each cell cluster in the T2D group. (A) B cells; (B) Monocytes/macrophages 1; (C) Monocytes 1; (D) Unknown cell type; (E) DCs; (F) Small pre-B cells; (G) Monocytes 2; (H) Monocyte progenitors; (I) T cells; (J) GMPs; (K) Basophils/eosinophils; (L) Pro-B cells; (M) Large pre-B cells; (N) NK cells; (O) Monocytes/macrophages 2; (P) Unknown cells.

**Figure. S6**

**
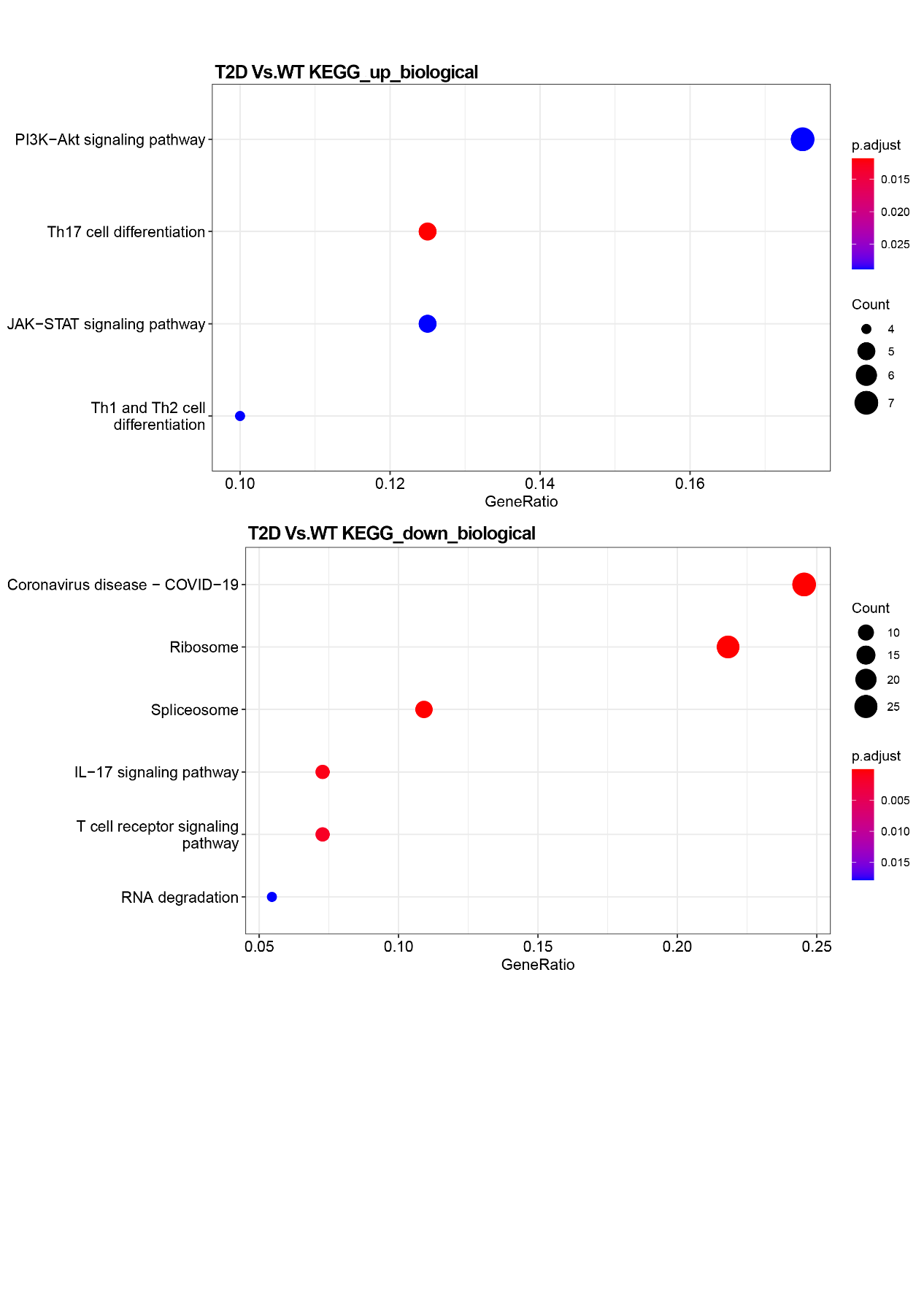
**

**Figure S6.** KEGG pathway analysis of up-regulated and down-regulated DEGs of T cells.

**Figure. S7**

**
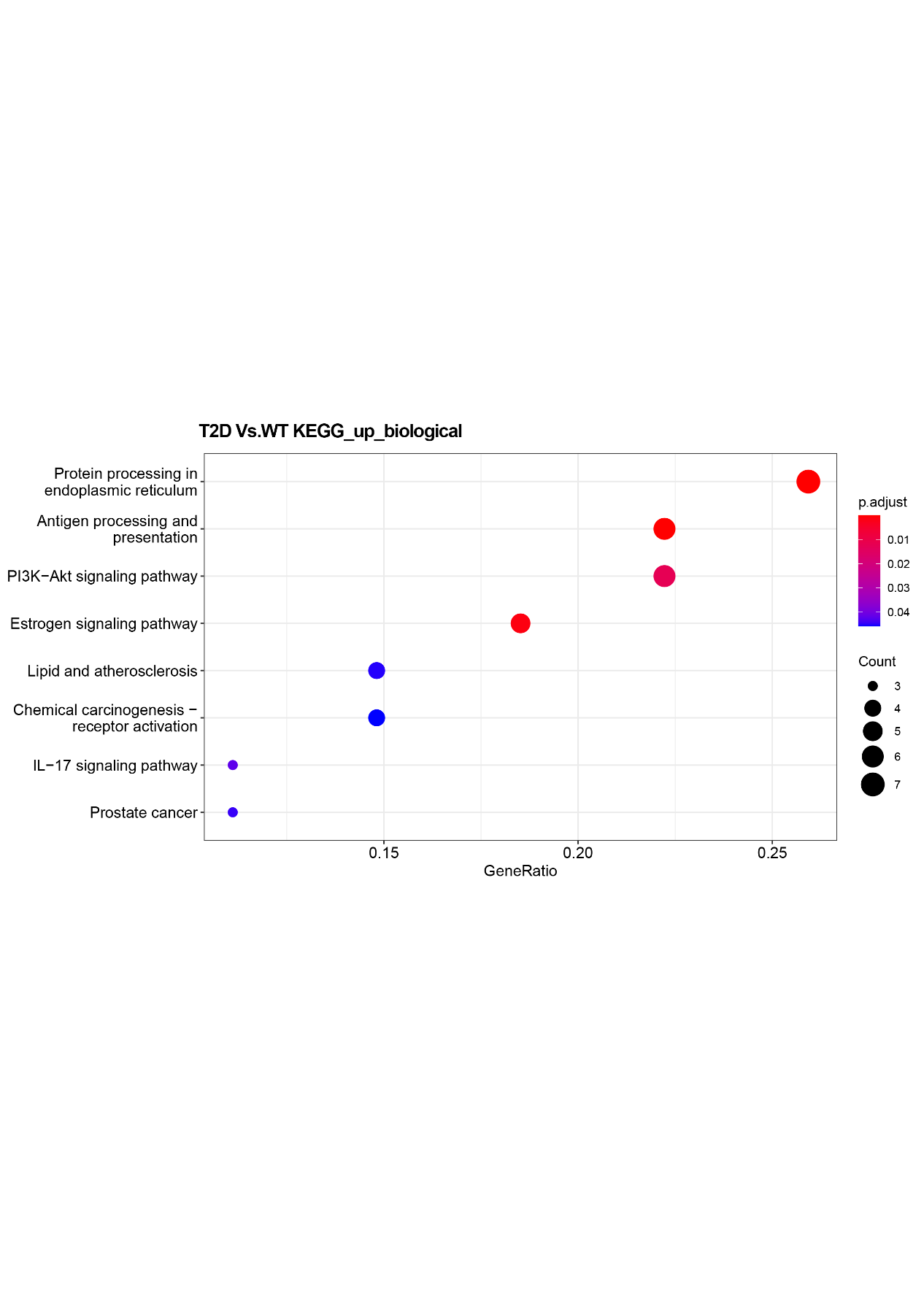
**

**Figure. S7** KEGG analysis of up-regulated DEGs in B cell.

**Figure. S8**


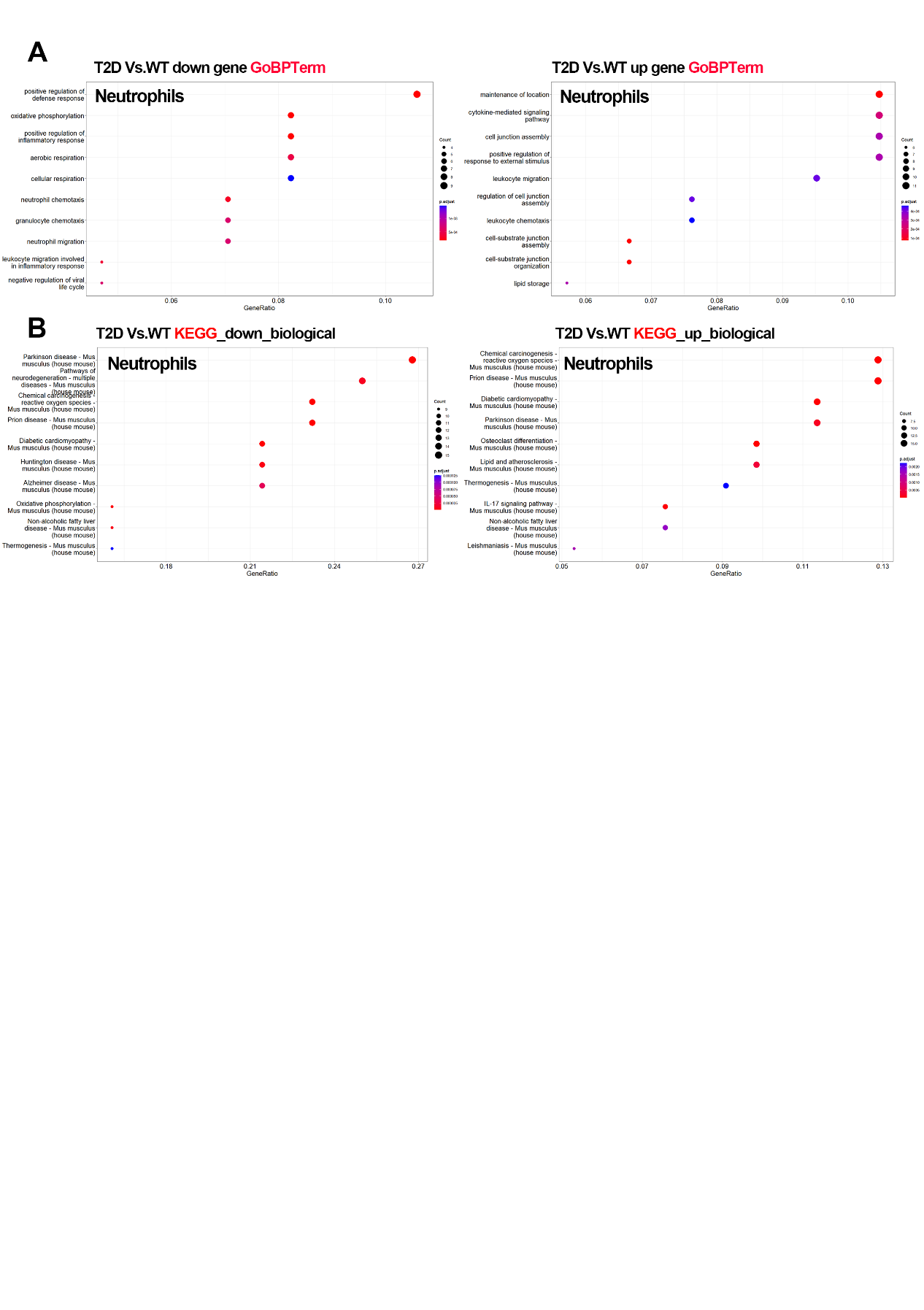


**Figure. S8** GO enrichment analysis **(A)** and KEGG pathway analysis **(B)** of the down- and up-regulated DEGs in neutrophils between WT and T2D mice.

**Figure. S9**


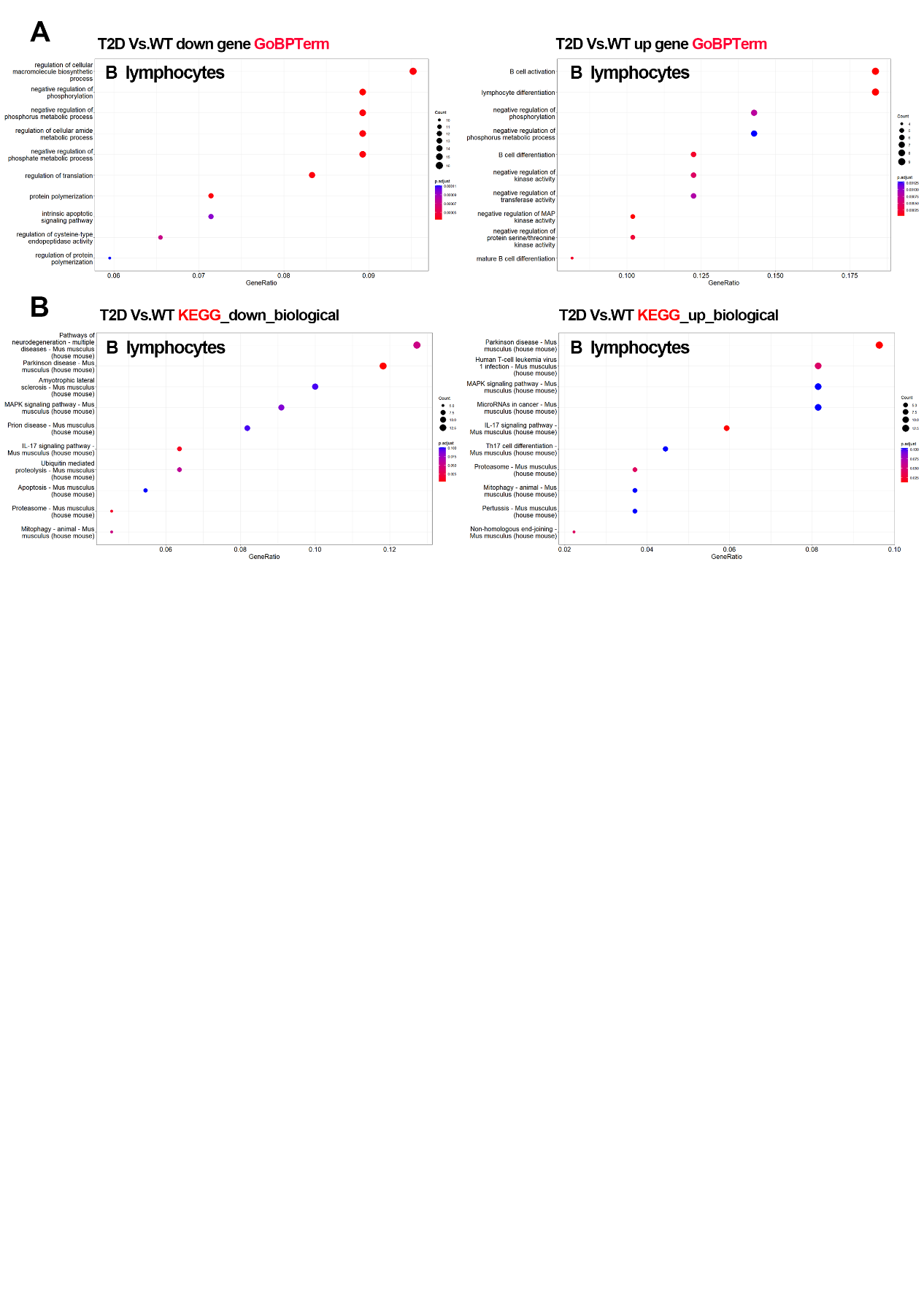


**Figure. S9** GO enrichment analysis **(A)** and KEGG pathway analysis **(B)** of the down- and up-regulated DEGs in B lymphocytes between WT and T2D mice.

**Figure. S10**


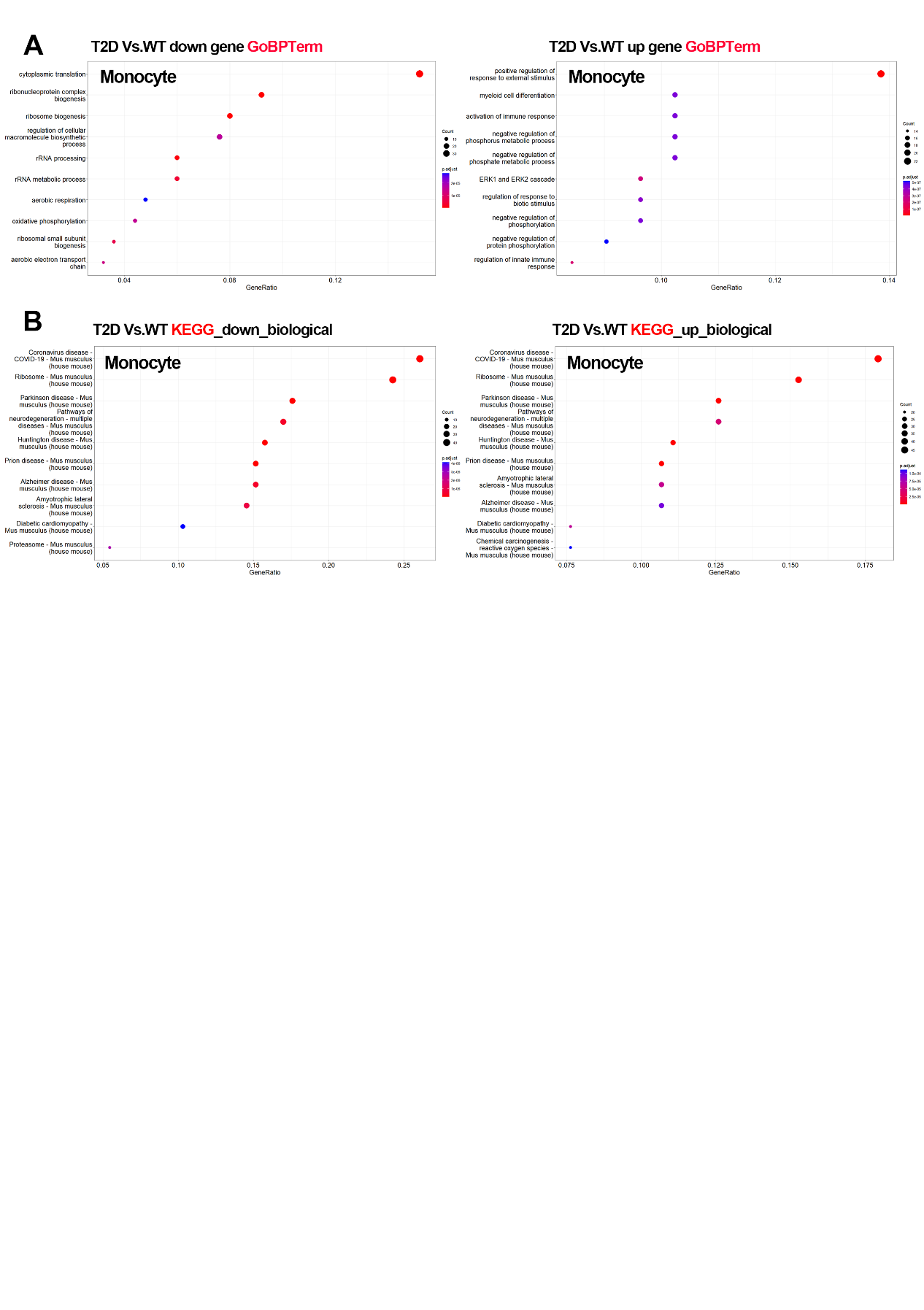


**Figure. S10** GO enrichment analysis **(A)** and KEGG pathway analysis **(B)** of the down- and up-regulated DEGs in monocytes between WT and T2D mice.

**Table. S1** The GO biological processes of cytokine/chemokine production and response to cytokines in the monocyte population.

| cluster | GObp | Description | levels | gene | number |
| --- | --- | --- | --- | --- | --- |
| Monocyte 1(Cluster 3) | GO:0050727 | regulation of inflammatory response | up | *Sod1*, *Lyn*, *Bst1*, *Fcgr1*, *Nfkbia*, *Ctsc*, *Nlrx1*, *Apoe*, *Sbno2*, *Grn*, *Cebpb*, *C3*, *Fcgr3* | 13 |
|  | GO:1990869 | cellular response to chemokine | down | *Ccl3*, *Ccl2*, *Dusp1*, *Rhoa*, *Cxcl2*, *Cx3cr1* | 6 |
|  |  |  |  |  |  |
| Monocytes 2 (Cluster 10) | GO:0050727 | regulation of inflammatory response | up | *Bst1*, *Sod1*, *Apoe*, *Lyn*, *Nfkbia*, *Sbno2*, *Fcgr1*, *Grn*, *C3*, *Fcgr3*, *Il16* | 11 |
|  |  | regulation of inflammatory response | down | *Ccl3*, *Gpx1*, *Cx3cr1*, *Lpl*, *Adrb2*, *Osm*, *Anxa1*, *Zfp36*, *S100a9*, *Cd24a*, *Ldlr*, *Trem2*, *Psma1*, *H2-T23*, *Pglyrp1* | 15 |
|  | GO:0032642 | regulation of chemokine production | up | *Lrp1*, *Il4ra*, *Syk*, *Il16* | 4 |
|  | GO:0032615 | interleukin-12 production | up | *Thbs1*, *Plcb1*, *Syk*, *Il16* | 4 |
|  | GO:0032733 | positive regulation of interleukin-10 production | up | *Bcl3*, *Stat3*, *Syk* | 3 |
|  | GO:0032612 | interleukin-1 production | down | *Ccl3*, *Cx3cr1*, *Lpl*, *Anxa1*, *Trem2*, *Tnfaip8* | 6 |
|  | GO:1990869 | cellular response to chemokine | down | *Ccl3*, *Ccl2*, *Cx3cr1*, *Dusp1*, *Cxcl2*, *Rhoa*, *Trem2*, *Cib1* | 8 |
|  |  |  |  |  |  |
| Monocytes/Macrophages 1(Cluster 1) | GO:0050727 | regulation of inflammatory response | up | *Bst1*, *Apoe*, *Sod1*, *Fpr2*, *Grn*, *Fcgr1*, *Lyn*, *Ace*, *Nfkbia*, *Fcgr2b*, *Sbno2* | 11 |
|  |  | regulation of inflammatory response | down | *Lpl*, *Ccl3*, *Cx3cr1*, *Zfp36*, *Osm*, *Adrb2*, *Alox5ap*, *Pglyrp1*, *Cd24a*, *Ldlr*, *Anxa1*, *Trem2*, *Socs3* | 13 |
|  | GO:1990869 | cellular response to chemokine | up | *Ccl3*, *Ccl2*, *Dusp1*, *Cx3cr1*, *Cxcl2*, *Padi2*, *Cib1*, *Trem2* | 8 |
|  | GO:0070670 | response to interleukin-4 | up | *Cd300lf*, *Xbp1*, *Hspa5*, *Parp14* | 4 |
|  | GO:0032612 | interleukin-1 production | down | *Lpl*, *Ccl3*, *Cx3cr1*, *Tyrobp*, *Anxa1*, *Trem2* | 6 |
|  | GO:0032635 | interleukin-6 production | down | *Lpl*, *Tyrobp*, *Klf2*, *Mbp*, *Cd24a*, *Cd84*, *Trem2* | 7 |
|  |  |  |  |  |  |
| Monocytes/  Macrophages 2 (Cluster 18) | GO:0050727 | regulation of inflammatory response | down | *Hyal2*, *Cx3cr1*, *Alox5*, *Ndfip1*, *Socs5*, *Tnfaip8l2*, *Pglyrp1*, *Anxa1*, *Sirpa*, *Tlr2*, *Abr*, *Gpsm3*, *Pparg*, *Pycard*, *Cd47*, *Socs3*, *Tlr6*, *Bap1*, *Il1b*, *Tnf*, *Ptger4*, *S100a9*, *Otulin*, *Pik3cg*, *Tbc1d23*, *Vamp8*, *Smad3* | 27 |
|  | GO:0032642 | regulation of chemokine production | down | *Havcr2*, *Socs5*, *Klf4*, *Sirpa*, *Tlr2*, *Hmgb1*, *Pycard*, *Egr1*, *Elane*, *Il1b*, *Tnf*, *Cd74*, *Map2k5* | 13 |
|  | GO:0032612 | interleukin-1 production | down | *Havcr2*, *Cx3cr1*, *Anxa1*, *Sirpa*, *Tlr2*, *Hmgb1*, *Usp50*, *Pycard*, *Egr1*, *Tlr6*, *P2rx7*, *Gsdmd*, *Il1b*, *Tnf*, *Ptger4*, *Serpinb1a*, *Hdac2*, *Il1r2*, *Smad3* | 19 |
|  | GO:0032637 | interleukin-8 production | down | *Hyal2*, *Anxa1*, *Klf4*, *Tlr2*, *Pycard*, *Elane*, *Tlr6*, *Lamtor5*, *Il1b*, *Tnf*, *Ptger4*, *Cd74*, *Map2k5* | 13 |
|  | GO:0032635 | interleukin-6 production | down | *Havcr2*, *Hyal2*, *Cd300ld*, *Socs5*, *Sirpa*, *Tlr2*, *Hmgb1*, *Pycard*, *Cd47*, *Tlr6*, *P2rx7*, *Il1b*, *Tnf*, *Tbc1d23*, *Cyba*, *Cd74* | 16 |
|  | GO:0070555 | response to interleukin-1 | down | *Hyal2*, *Cd40*, *Anxa1*, *Sirpa*, *Pycard*, *Cd47*, *Ccl2*, *Egr1*, *Il1b*, *Il1r2* | 10 |
|  | GO:0032621 | interleukin-18 production | down | *Tlr2*, *Usp50*, *Tnf* | 3 |

**Table. S2** Primers used in qPCR

| Gene | Forward primer sequence (5’-3’) | Reverse primer sequence (3’-5’) |
| --- | --- | --- |
| Fosb | ACCTGTCTTCGGTGGACTCCTTC | AAGATCCTGGCTGGTTGTGATTGC |
| Relb | CTACAATGCTGGCTCCCTGAAGAAC | ATGTCCCTGCTGGTCCCGATAG |
| Il1b | CACTACAGGCTCCGAGATGAACAAC | TGTCGTTGCTTGGTTCTCCTTGTAC |
| Map2k1 | TGGAGATGGCAGTTGGGAGATACC | TCCTTCCACATGGCATCCAAACAG |
| Map3k7 | CTCGTCCTCCTCCTCGTCTTCTG | TCTTCCGACAACCTCTTCCACCTC |
| Pik3cg | GACCTGTGCCTTCTGCCTTACG | CCCACTGTGCTTTGCTGAATTTGAG |
| Ppp3r1 | TTGAGCGTGGAAGAGTTCATGTCTC | CTTCTCCGTTGCCGTCTGTGTC |
| Sirpb1a | GTCCTGCTGTTGATCCTGCTTCTG | CCACAGGGAGGAGGGATGTCAC |
| Socs3 | GACCAAGAACCTACGCATCCAGTG | GCACCAGCTTGAGTACACAGTCG |
| Tgfbr2 | ATCTGTGAGAAGCCGCATGAAGTC | AGAGTGAAGCCGTGGTAGGTGAG |
| Tnf | CGCTCTTCTGTCTACTGAACTTCGG | GTGGTTTGTGAGTGTGAGGGTCTG |
| Rankl | ATGGAAGGCTCATGGTTGGATGTG | TGGCAGCATTGATGGTGAGGTG |
| Rank | TGAGCCTCCGAGCAGAACTGAC | CTGCCTGTGTAGCCATCTGTTGAG |
| OPG | TGTCCCTTGCCCTGACCACTC | CCTCACACTCACACACTCGGTTG |
| Traf6 | AGGAATCACTTGGCACGACACTTG | TCGCACGGACGCAAAGCAAG |
| Ctsk | GGCAGGGTCCCAGACTCCATC | TGAAAGCCCAACAGGAACCACAC |
| Itgb3 | GGAAGGCTGGCAGGCATTGTC | ATGGTAGTGGAGGCAGAGTAGTGG |
| Actb | GTGACGTTGACATCCGTAAAGA | GCCGGACTCATCGTACTCC |
